# TRPV1 alleviates APOE4-dependent microglial antigen presentation and T cell infiltration in Alzheimer's disease

**DOI:** 10.1186/s40035-024-00445-6

**Published:** 2024-10-29

**Authors:** Jia Lu, Kexin Wu, Xudong Sha, Jiayuan Lin, Hongzhuan Chen, Zhihua Yu

**Affiliations:** 1 Department of Pharmacology and Chemical Biology, Shanghai Jiao Tong University School of Medicine, Shanghai, 200025 China; 2 Shanghai University of Traditional Chinese Medicine, Shanghai, 201203 China

**Keywords:** APOE4, Cholesterol, TRPV1, Microglia, Tau

## Abstract

**Background:**

Persistent innate and adaptive immune responses in the brain contribute to the progression of Alzheimer’s disease (AD). *APOE4*, the most important genetic risk factor for sporadic AD, encodes apolipoprotein E4, which by itself is a potent modulator of immune response. However, little is known about the immune hub that governs the crosstalk between the nervous and the adaptive immune systems. Transient receptor potential vanilloid type 1 (TRPV1) channel is a ligand-gated, nonselective cation channel with Ca^2+^ permeability, which has been proposed as a neuroprotective target in AD.

**Methods:**

Using Ca^2+^-sensitive dyes, dynamic changes of Ca^2+^ in microglia were measured, including exogenous Ca^2+^ uptake and endoplasmic reticulum Ca^2+^ release. The mRFP-GFP-tagged LC3 plasmid was expressed in microglia to characterize the role of TRPV1 in the autophagic flux. Transcriptomic analyses and flow cytometry were performed to investigate the effects of *APOE4* on brain microglia and T cells from *APOE*-targeted replacement mice with microglia-specific *TRPV1* gene deficiency.

**Results:**

Both *APOE4* microglia derived from induced pluripotent stem cells of AD patients and *APOE4*-related tauopathy mouse model showed significantly increased cholesterol biosynthesis and accumulation compared to their *APOE3* counterparts. Further, cholesterol dysregulation was associated with persistent activation of microglia and elevation of major histocompatibility complex II-dependent antigen presentation in microglia, subsequently accompanied by T cell infiltration. In addition, TRPV1-mediated transient Ca^2+^ influx mitigated cholesterol biosynthesis in microglia by suppressing the transcriptional activation of sterol regulatory element-binding protein 2, promoted autophagic activity and reduced lysosomal cholesterol accumulation, which were sufficient to resolve excessive immune response and neurodegeneration in *APOE4*-related tauopathy mouse model. Moreover, microglia-specific deficiency of *TRPV1* gene accelerated glial inflammation, T cell response and associated neurodegeneration in an *APOE4*-related tauopathy mouse model.

**Conclusions:**

The findings provide new perspectives for the treatment of *APOE4*-dependent neurodegeneration including AD.

**Supplementary Information:**

The online version contains supplementary material available at 10.1186/s40035-024-00445-6.

## Introduction

Apolipoprotein E (ApoE) is a 34-kDa lipid-binding glycoprotein that transports cholesterol and phospholipids across different cell types in the brain to maintain lipid metabolism. Among the ε2–ε4 polymorphic alleles, the ε4 allele of *APOE* gene (*APOE4*) is the strongest genetic risk factor for late-onset Alzheimer’s disease (AD). Altered immunological milieux, especially activated microglia and T cell responses, has been demonstrated in AD patients carrying *APOE4* and in an AD mouse model expressing human *APOE4* [1–3]. However, it remains unclear how ApoE4 impacts the immune hub that governs the crosstalk between the nervous and the adaptive immune systems.

Global transcriptomic analyses have revealed *APOE*4-driven cholesterol metabolic dysregulation in microglia, which may contribute to the innate immune response in AD [4, 5]. Recent studies in humanized mouse models and on human induced pluripotent stem cells (hiPSCs) from AD patients carrying the *APOE4* variant showed enhanced de novo cholesterol synthesis and increased intracellular cholesterol content in microglia [4, 6, 7]. Cholesterol is an intrinsic component of lipid rafts, specialized cell membrane microdomains for organizing signaling molecules such as major histocompatibility complex II (MHC II). In the peripheral immune system, cholesterol accumulation enhances the function of MHC II-dependent antigen presentation in dendritic cells [8]. However, the underlying molecular mechanisms for the immunomodulatory role of APOE4 in AD pathogenesis have not been fully elucidated.

Transient receptor potential vanilloid 1 (TRPV1), also known as the capsaicin receptor, is a ligand-gated nonselective cation channel with Ca^2+^ permeability, and has neuroprotective effects against neurodegenerative diseases [9–12]. TRPV1 is functionally expressed and regulates diverse microglial functions, including inflammation, phagocytosis and cytokine production [10, 13, 5]. TRPV1 activation leads to cholesterol efflux via upregulation of ATP-binding cassette (ABC) transporters in macrophages through liver X receptor α-dependent regulation of transcription in atherosclerosis. TRPV1 agonist capsaicin, the active component in hot chili peppers, attenuates foam cell formation by promoting autophagy in oxidized low-density lipoprotein-treated vascular smooth muscle cells [14]. Our previous study reported that administration with capsaicin significantly relieves microglial phagocytosis of synapses through downregulating the expression of neuronal major histocompatibility complex I to protect against neurodegeneration in *APOE4* mice on high-fat diet. We also observed that ApoE4 increases lipid droplet accumulation in microglia, which leads to enhanced MHC II-dependent antigen presentation and peripheral adaptive immune cell infiltration in the mouse brain [5]. However, the protective effect of capsaicin on this pathologic phenomenon and its potential mechanism remain largely unknown.

Here, we found that *APOE4*-induced endoplasmic reticulum (ER) stress, specifically resulting from ER Ca^2+^ depletion, promotes the sterol regulatory element-binding protein (SREBP) 2 activation of the microglial cholesterol biosynthesis pathways. Cholesterol accumulation is associated with the activated state of microglia and elevation of MHC II-dependent antigen presentation in microglia, subsequently accompanied by T cell infiltration. Capsaicin increases the ER Ca^2+^ levels in microglia to attenuate the *APOE4*-induced activation of SREBP2 and antigen presentation, and reversed the impairment of microglial autophagy in vitro. Capsaicin also attenuated excessive immune response and neurodegeneration in an *APOE4*-related tauopathy mouse model. Furthermore, microglia-specific *TRPV1* gene deficiency accelerates glial inflammation, T cell response and associated neurodegeneration. These findings may provide new perspectives for the treatment of *APOE4*-dependent neurodegeneration including AD.

## Materials and methods

### Mice

TRPV1^*flox/flox*^; Cx3cr1^Cre^ mice with microglia-specific conditional knockout of TRPV1 channels have been described previously [9]. Human *APOE3*- and *APOE4*-targeted replacement homozygous mice were gifted from Prof. Yu Qiu (Department of Pharmacology and Chemical Biology, Shanghai Jiao Tong University School of Medicine, Shanghai, China). TRPV1^*flox/flox*^ mice were bred to human *APOE3*- and *APOE4*-targeted replacement homozygous mice to generate TRPV1^*flox/flox*^/APOE3 mice (denoted E3 mice) and TRPV1^*flox/flox*^/APOE4 mice (denoted E4 mice). TRPV1^*flox/flox*^; Cx3cr1^Cre^ mice were intercrossed with human *APOE3* or *APOE4* targeted replacement homozygous mice to generate TRPV1^*flox/flox*^; Cx3cr1^Cre^/APOE3 mice (denoted TRPV1^−/−^/E3 mice) and TRPV1^*flox/flox*^; Cx3cr1^Cre^/APOE4 mice (denoted TRPV1^−/−^/E4 mice).

Four-week-old male and female TRPV1^−/−^/E4 mice were intraperitoneally administered with tamoxifen (sc-208414; Santa Cruz Biotechnology, Santa Cruz, CA) at a dose of 75 mg/kg per day for 5 consecutive days. The mice were group housed (four to five mice per cage) at room temperature (22 ± 1 °C) under a 12-h light/dark cycle. All animal experimental procedures were approved by the Animal Experimentation Ethics Committee of Shanghai Jiao Tong University School of Medicine (Shanghai, China).

### RNA-seq data acquisition and analysis

The raw RNA-seq data on population microglia derived from hiPSCs of AD patients with different APOE genotypes were downloaded from Gene Expression Omnibus [15] with accession number GSE190187 [4]. Differentially expressed genes were identified with the DESeq software after excluding genes with zero read counts across all samples. Gene Set Enrichment Analysis (GSEA) was performed using hallmark, curated and ontology gene sets available from the UCSD and the Broad Institute (https://www.gsea-msigdb.org/gsea/index.jsp).

### Preparation of paired helical filament (PHF) from tau protein

Preparation of PHF was performed as described previously [16, 17]. Recombinant human microtubule-associated protein tau monomers were purchased from Sangon (C521979; Sangon Biotech, Shanghai, China). For fibril assembly, tau monomers (50 μM) in phosphate-buffered saline (PBS) were incubated with 12.5 μM heparin lithium salt (A690013; Sangon Biotech), 2 mM dithiothreitol (DTT; B645939; Sangon Biotech) and a protease inhibitor cocktail (P1025; Beyotime Institute of Biotechnology, Shanghai, China) for 2 weeks at 37 °C. Fibril assembly was induced by the addition of heparin. Reducing conditions were maintained by addition of 1 mM DTT every 24 h, because the disulfide bond formation in the four-repeat domain of tau decreases the rate of polymerization. After assembly, excess heparin and DTT were removed by dialysis against PBS for 24 h. Tau PHF was divided into aliquots and stored at −80 °C. Aggregates were sonicated immediately before use. The assembly of tau PHF was verified by transmission electron microscopy (TEM).

#### TEM

The morphology of PHF and sonicated PHF was verified by TEM [17]. Specimens were absorbed onto 75-mesh formvar/carbon-coated copper grids. The grids were washed with water and stained with 2% uranyl acetate. Excess solution was then blotted with filter paper, and the grids were allowed for air dry. Images were captured on an H-7650 transmission electron microscope (Hitachi, Tokyo, Japan).

### Stereotaxic brain injection

AAV-hTau and control AAV-GFP were purchased from OBio Biologic Technology Co. Ltd (Shanghai, China). The titer of AAV-hTau was 3.14 × 10^12^ v.g./ml, and that of AAV-GFP was 4.09 × 10^13^ v.g./ml. The efficiency of in vivo overexpression was examined by fluorescence 1 month after injection of the virus into the hippocampal CA3 region in mice. E3 mice, E4 mice, TRPV1^−/−^/E3 mice and TRPV1^−/−^/E4 mice (2–3 months old) were randomly allocated to experimental groups. For stereotaxic brain injection, mice were anesthetized with 1% pentobarbital sodium by intraperitoneal injection and then positioned in a stereotaxic instrument (RWD Life Science Co., Ltd., Shenzhen, China). Subsequently, 1 μl of the virus was bilaterally injected into the hippocampal CA3 region (AP –2.0, ML ± 1.5, DV –2.0) at a rate of 0.2 µl/min with an injection pump [18]. The injection needle remained in the target region for 5 min after the end of injection to allow for diffusion. Finally, the cutaneous wound was sutured.

### Capsaicin administration

Mice were allowed to recover for 1 week after the stereotaxic injection, and then treated with capsaicin (T1062; TargetMol, Shanghai, China) at 1 mg/kg (intraperitoneal injection) once daily for 30 days. Afterward, the mice were subjected to behavioral tests, and brain tissues were collected for magnetic bead sorting, flow cytometry and histological analyses.

### Behavioral tests

To evaluate the impact of microglial TRPV1 channels on learning and memory in murine AD model, open field test, Y maze test, and Morris water maze (MWM) test were conducted after capsaicin treatment. All behavioral experiments were conducted in a blind manner.

#### Open field test

The open field test was performed as previously described [19]. Briefly, an experimental mouse was placed in an empty chamber with clear sidewall (40 cm × 40 cm × 40 cm) and allowed to move freely in the chamber for 10 min. Locomotor activity was tracked with the Noldus Ethovision system (Noldus Information Technology, Wageningen, Netherlands).

#### Y maze test

Spatial working memory was measured by Y maze test as previously described [20]. The Y maze was a three-arm maze with an angle of 120 degrees between each two arms (50 × 18 × 35 cm^3^). The test consisted of two trials separated by an interval of 24 h. On the first day, a mouse was placed into the Y maze for 10 min to adapt to the environment. On the next day, the mouse was placed into the center of the maze and allowed to explore freely through the maze for 10 min. Video tracking was performed using the Noldus Ethovision system, and the number of total arm entries and the order of entries into the arms were recorded. Spontaneous alternation was counted as the number of alterations when the mouse entered the three different arms consecutively divided by the number of total arm entries multiplied by 100%.

#### MWM test

The MWM test was performed as described [9]. A round white pool (120-cm diameter, 50-cm depth) with a tracking and analysis system (Noldus Information Technology) was used for the test. An escape platform (4.5 cm in diameter) was submerged 1 cm below the water surface. For spatial learning, mice were trained to find the hidden escape platform in water maze with 4 trials (60 s maximum; interval 30 min) per day for 5 consecutive days. In each trial, a mouse was placed into the pool from one of the four quadrants facing the wall and the trial ended when it climbed on the platform. Mouse was guided to the platform and stayed there for 10 s if it failed to locate the platform within 60 s. The time used to find the platform (latency) was recorded each day. Two probe trials were carried out on the sixth day after removing the platform. The number of times the mouse swam across the platform site, the total distance traveled, the time and the traveling distance in the previous platform quadrant were recorded automatically for analysis.

### Mononuclear cell isolation and flow cytometry

Mononuclear cells were isolated from the hippocampus and cortex in mice according to previously published methods [21–23]. Mice were anesthetized and then perfused with 1 × Hanks’ balanced salt solution (C0218; Beyotime Institute of Biotechnology). To obtain single-cell suspensions, the hippocampus and cortex were dissected from the brain and digested with 0.5 mg/ml collagenase IV (40510ES60; Yeasen, Shanghai, China) and 0.2 mg/ml deoxyribonuclease I (DN25; Sigma-Aldrich, St. Louis, MO) in serum-free Dulbecco’s modified Eagle’s medium (DMEM) (L10KJ; BasalMedia, Shanghai, China) at 37 °C. After myelin was removed by the 30% Percoll gradient (CP8331; Coolaber, Beijing, China) at 500 × *g* for 30 min, the total cell pellet was resuspended in DMEM and passed through a 70-μm filter. Dissociated cells were surface stained with fluorescently conjugated antibodies CD11b-APC (clone M1/70; BioLegend, San Diego, CA, USA), CD45-APC/Fire 750 (clone 30-F11; BioLegend), MHC II-PE (clone M5/1114.15.2; BioLegend), CD8a-PE/Cy7 (clone53-6.7; BioLegend), TCRβ-PerCP/Cy5.5 (clone H57-579; BioLegend) and CD4-Alexa Fluor 700 (clone RM4-4; BioLegend). Samples were analyzed by flow cytometry with a BD LSRFortessa X-20 cell analyzer (BD Bioscience, San Jose, CA) and the FlowJo software (FlowJo, LLC, OR).

### Immunofluorescence and quantitative analysis

Brain sections containing the hippocampus were blocked and permeabilized with 10% goat serum in PBS with 0.3% Triton X-100 for 1 h at room temperature. Afterward, the sections were labeled with the following primary antibodies at 4°C overnight: mouse anti-NeuN (1:1000; ab279296, Abcam, Cambridge, UK), rabbit anti-MAP2 (1:200; 8707, Cell Signal Technology, Danvers, MA), rabbit anti-phospho-tau AT8 (p-S202/T205) (1:200; GB113883, Sevicebio Technology, Wuhan, China), rabbit anti-phospho-tau AT180 (p-T231) (1:200; NB100-82249, Novus Biological, Littleton, CO), rabbit anti-PSD95 (1:400; 3409, Cell Signal Technology), mouse anti‐Iba1 (1:200; GB12105, Sevicebio Technology), rabbit anti‐Iba1 (1:500; 019‐19741, Wako Pure Chemical Industries, Osaka, Japan), rat anti‐Iba1 (1:100; ab283346, Abcam), rat anti‐CD68 (1:1000, ab53444, Abcam), rabbit anti-phosphorylated eukaryotic initiation factor-2α (p-eIF2α) (Ser51) (1:50; 3398, Cell Signal Technology), mouse anti‐SREBP2 (1:50; sc-1352, Santa Cruz Biotechnology), rat anti‐MHC II (1:500; 107601, BioLegend), rabbit anti-‧C–C Motif Chemokine Ligand 8 (CCL8) (1:100; bs-1985R, Bioss, Beijing, China), rabbit anti-CCR1 (1:50; A18341, ABclonal, Wuhan, China), rat anti‐CD4 (1:100; 100505, BioLegend), and rat anti‐CD8a (1:50; 100701, BioLegend). The sections were next incubated with Alexa Fluor 647 goat anti-rat (1:500; ab150159, Abcam), Cy3 goat anti-rabbit (1:200; GB21303, Sevicebio Technology) and Cy3 goat anti-mouse (1:200; GB21301, Sevicebio Technology). Sections were washed once and stained with 1 μg/ml BODIPY 493/503 in PBS for 10 min to visualize lipid droplets, and incubated with DAPI (1:1000; D9542, Thermo Fisher Scientific, Waltham, MA) for 10 min at room temperature for nuclear counterstaining. Images were captured using a TCS SP8 confocal laser scanning microscope (Leica Microsystems, Nussloch, Germany) and subsequently analyzed with the Fiji software (NIH, Bethesda, MD).

### Three-dimensional (3D) reconstruction of microglia

Confocal image stacks of Iba1^+^ or BODIPY^+^ microglia were reconstructed and analyzed using the Imaris software (version 10.1.0; Bitplane Imaris). Briefly, the filament tracer was used to identify Iba1^+^ microglia with their dendritic processes for morphology quantification, and BODIPY signal in individual 3D reconstructed microglia was measured using the Local Contrast function of the Imaris software.

### Adult mouse microglial isolation and RNA extraction

Microglial isolation from adult mouse brains was performed as previously described [9]. Briefly, mice were anesthetized and then intracardially perfused with ice-cold PBS, and the cortex and hippocampus were dissected. The tissues were mechanically and enzymatically digested with papain (A501612; Sangon Biotech) to obtain cell suspensions, then filtered through a 70-µm cell strainer to remove any debris. To enrich the microglial population, we incubated the single cell suspensions at 4 °C for 40 min with biotin-conjugated anti-CD11b (13-0112-82; eBioscience, San Diego, CA); subsequently, magnetic sorting was performed with Dynabeads™ Biotin Binder (11047; Thermo Fisher Scientific) to obtain CD11b^+^ microglia. Total RNA was extracted from the CD11b^+^ microglia with a RNeasy Micro Kit (74004; Qiagen, Germantown, MD) according to the manufacturer’s instructions. RNA quality was assessed with a NanoDrop 2000 instrument (Thermo Fisher Scientific) to validate RNA concentration and purity, and agarose gel electrophoresis or an Agilent 2100 Bioanalyzer (Agilent Technologies, Santa Clara, CA) was used to verify RNA integrity.

### RNA sequencing

The SMARTer Ultra Low Input RNA Kit was used to prepare cDNA using 50 ng total RNA extracted from CD11b^+^ microglia. The first strand of cDNA was synthesized by using Oligo (dT) as the primer, which could combine with the mRNA with a poly A structure, and subsequently, the second strand of cDNA was synthesized with random primers. The double-strand cDNA was purified and underwent end reparation and 3′-end single nucleotide A (adenine) addition. Libraries were constructed from the amplified cDNA by treatment with Tn5 transposase and checked for quality on Agilent 2100 Bioanalyzer. Multiplexed DNA libraries were sequenced on the Illumina sequencing platform with the strategy of PE150. The FASTQ raw data were trimmed for adapters and preprocessed to remove low-quality sequences, and clean reads were mapped to the mouse reference genome using HISAT2 (http://ccb.jhu.edu/software/hisat2/index.shtml). HTseq (v0.9.1) was used to produce raw read counts per gene, and gene expression counts for each sample were quantified as normalized Fragments Per Kilo bases per Million fragments. The DESeq software (v1.38.3) was used to perform differential expression analysis according to the screening conditions: absolute value of log2 (fold change) larger than 1 and significance *P* value < 0.05. Gene enrichment analyses were performed using topGO (v2.50.0), clusterProfiler (v4.6.0) and GSEA (v4.1.0) to summarize the biological processes (BPs) of the differential expression genes. Volcano plots were produced using the R ggplot2 package.

### BV2 cell culture

The murine microglial BV2 cell line was originally purchased from the Cell Bank of the Chinese Academy of Sciences. Cells were cultured in DMEM, supplemented with 10% FBS (S711-001S; Shuangru Biotechnology, Shanghai, China), 1% penicillin/streptomycin (C0222; Beyotime Institute of Biotechnology) and 2 mM L-glutamine (C0212; Beyotime Institute of Biotechnology) under standard culture conditions (5% CO_2_ at 37 °C). Cells were sub-cultured every 3–4 days at 1:10 split ratio.

### Stimulation of BV2 cells

Cholesterol enrichment of BV2 cells was achieved by cholesterol transport through ApoE3 (350–02; Pepro Tech, Rocky Hill, NJ) or ApoE4 (350–04; Pepro Tech); 10% FBS was added to provide a source of cholesterol to ApoE, because purified *Escherichia coli*-derived ApoE3 and ApoE4 were devoid of cholesterol [24, 25]. BV2 cells were incubated with ApoE3 or ApoE4 (4 μg/ml) containing 10% fetal bovine serum (FBS) for 6 h, then washed with PBS, and further incubated with culture medium for 4 h. The contents of cholesterol and triglyceride were measured by labeling the cells with Filipin III (abs42018484; Absin Bioscience, Shanghai, China) and BODIPY 493/503 (D3922; Thermo Fisher Scientific). The BV2 cells were subsequently exposed to 1 μg/ml PHF for 24 h. For treatment experiments, BV2 cells were pretreated with 1 μM capsaicin to activate the TRPV1 channel for 30 min, and then 1 μg/ml PHF was added.

### Cholesterol and lipid in vitro staining and analysis

BV2 cells were stained with Filipin III or BODIPY 493/503 according to the manufacturer’s instructions. BV2 cells were fixed in 4% paraformaldehyde at room temperature for 20 min after specific treatments, then washed three times with PBS and incubated with 50 μg/ml Filipin III in PBS containing 10% FBS for 2 h to assess cholesterol content. BV2 cells were fixed and labeled with 1 μg/ml BODIPY 493/503 in PBS for 10 min, then stained with DAPI to visualize lipid droplet accumulation. Fluorescence images were photographed (40 × magnification) under a TCS SP8 confocal laser scanning microscope and analyzed with the ImageJ software. For flow cytometry analysis, BV2 cells were collected and stained with 0.5 μg/ml BODIPY 493/503 in PBS for 10 min at 37 °C, then analyzed by flow cytometry with an Attune NxT flow cytometer (Thermo Fisher Scientific) and the FlowJo software.

### LysoTracker staining

For intracellular lysosome and cholesterol co-localization, microglia were incubated with LysoTracker Red (C1046; Beyotime Institute of Biotechnology) at a final concentration of 50 nM in the dark at 37 °C for 30 min. Cells were washed twice with PBS after removing dye-containing media, and then fixed with 4% paraformaldehyde for 10 min. Filipin III staining was performed as described above. Images were obtained with the TCS SP8 confocal laser scanning microscope and analyzed with the ImageJ software.

### Immunofluorescence of BV2 cells

Surface distribution of MHC II molecules was performed as previously described [8]. Microglia were fixed with 4% paraformaldehyde at room temperature for 10 min, blocked in PBS containing 5% bovine serum albumin (K35-001; PAA Laboratories, Pasching, Austria), and then stained with FITC-conjugated cholera toxin B subunit (CTxb-FITC, 8 μg/ml; abs80003; Absin Bioscience, Shanghai, China) and PE-conjugate anti-MHC Class II I-A/I-E (MHC II-PE, 2 μg/ml; 107608; BioLegend) at 4°C for 30 min. The cells were then stained with DAPI. Fluorescent images were captured using a TCS SP8 confocal laser scanning microscope equipped with a 63 × 1.4 NA objective and analyzed by the ImageJ software.

Immunofluorescence staining of cultured cells was performed as previously described [9]. BV2 cells were fixed and blocked in 10% goat serum containing Triton X-100, and then incubated with mouse anti‐SREBP2 (1:50, sc-1352; Santa Cruz Biotechnology), mouse anti-ATP-binding cassette transport protein A1 (ABCA1) (1:100, ab66217; Abcam), rabbit anti-Rab11 (1:200, AF7851; Beyotime Institute of Biotechnology) or rabbit anti-LMAP2 (1:200, AF1036; Beyotime Institute of Biotechnology) at 4 °C overnight. After washes in PBS, cells were stained with Alexa Fluor 647 goat anti-mouse (1:1000; A32728, ThermoFisher Scientific) or Cy3 goat anti-rabbit (1:200; GB21303, Sevicebio Technology) for 1 h, and then stained with DAPI. Fluorescent images were captured using a TCS SP8 confocal laser scanning microscope and analyzed by the ImageJ software.

### Measurement of Ca^2+^ fluorescence

Intracellular calcium was measured as previously described [26]. After the indicated treatments, BV2 cells were washed once in Ca^2+^-free buffer comprising 1 × Hanks’ balanced salt solution containing 0.04% Pluoronic-F127 (ST501; Beyotime Institute of Biotechnology) and 2 mM probenecid (T045T; TopScience, Shanghai, China). Then the cells were stained with calcium fluorescent probe Mag-Fluo-4 AM (3 μM; 20401, AAT Bioquest, Sunnyvale, CA) or Fluo-4 AM (1.5 μM; S1060, Beyotime Institute of Biotechnology) for 30 min at 37 °C in the dark for the ER or cytosolic Ca^2+^, respectively. After incubation, BV2 cells were washed once to remove the extracellular probe, and fluorescence signals were measured at different time points with a FlexStation 3 Multi-Mode Microplate Reader (Molecular Devices, Sunnyvale, CA). The release of ER Ca^2+^ store was detected after stimulation by 1 μM thapsigargin (Tg) (T9033; Sigma-Aldrich); after the Ca^2+^ concentration became stable, 2 mM CaCl_2_ (A501330; Sangon Biotech) was added to induce Ca^2+^ uptake.

### Quantitative real-time PCR

Total RNA was isolated from BV2 cells using TRIzol reagent (R0016; Beyotime Institute of Biotechnology) and RNA concentration was measured with a BioDrop spectrophotometer (Biochrom Ltd, Cambridge, UK). RNA Samples were reverse transcribed to cDNA using a cDNA synthesis kit (6210A; Takara, Otsu, Japan). Quantitative real-time PCR was performed with BeyoFast™ SYBR Green qPCR Mix (D7265; Beyotime Institute of Biotechnology) using the LightCycler 480II (Roche Applied Science, Mannheim, Germany). Primer sequences are shown in Table S1. The mRNA expression of target genes was normalized to *GAPDH* and calculated using the 2^−ΔΔCt^ method.

### Cytoplasmic and nuclear protein extraction

Nuclear and cytoplasmic fractions of proteins were obtained with a Nuclear and Cytoplasmic Protein Extraction Kit (P0027; Beyotime Institute of Biotechnology). Briefly, 1.5 × 10^6^ BV2 cells were collected and resuspended in 150 μl of cytoplasmic protein isolation solution A containing phenylmethanesulfonyl fluoride (PMSF) (ST-057, Beyotime Institute of Biotechnology), broken by vigorous vortexing and homogenized on ice for 15 min. Next, 7.5 μl of cytoplasmic protein isolation solution B was added and mixed by vortexing, and samples were centrifuged at 15,000 × *g* for 5 min at 4 °C. The supernatant fraction was collected as cytoplasmic proteins. The nuclear pellet was resuspended in 50 μl nuclear protein isolation solution containing PMSF, homogenized on ice for 30 min and centrifuged at 15,000 × *g* for 10 min at 4 °C. The supernatant fraction was collected as nuclear proteins.

### Western blotting

Western blotting analysis was performed as previously described [9]. BV2 cells were prepared in radioimmunoprecipitation lysis buffer (P0013B, Beyotime Institute of Biotechnology) containing PMSF. Lysates containing equal amounts of protein were resolved by 8% or 10% (*w/v*) sodium dodecyl sulfate–polyacrylamide gels and transferred to the polyvinylidene difluoride membrane. The membrane was blocked in 5% milk solution at room temperature for 1 h, and then probed with the primary antibodies overnight at 4 °C. The primary antibodies were as follows: rabbit anti-ApoE (1:1000; AF1921, Beyotime Institute of Biotechnology), rabbit anti-GRP78 (1:1000; AF0171, Beyotime Institute of Biotechnology), rabbit anti-p-eIF2α (Ser51) (1:1000; 3398, Cell Signal Technology), mouse anti‐SREBP2 (1:100; sc-1352, Santa Cruz Biotechnology), rabbit anti-ABCA1 (1:1000; AF6114, Beyotime Institute of Biotechnology), rabbit anti-ATP Binding Cassette Subfamily G Member 1 (ABCG1) (1:1000; AF1588, Beyotime Institute of Biotechnology), rabbit anti-Histone H4 (1:1000; AF7107, Beyotime Institute of Biotechnology) and β-actin (1:1000; 3700S, Cell Signal Technology). The membranes were then incubated with peroxidase-conjugated anti-mouse (1:1000; A0216, Beyotime Institute of Biotechnology) or anti-rabbit (1:1000; A0208, Beyotime Institute of Biotechnology) IgG at room temperature for 1 h. Immunoreactive bands were detected with an enhanced chemiluminescent substrate (36222ES76, Yeasen) and the Image Studio Lite Ver 5.2 software (LI-COR Biosciences, Lincoln, NE).

### Detection of reactive oxygen species (ROS)

After the indicated treatments, microglia were incubated with 10 μM 2′,7′-dichlorofluorescein diacetate (S0033; Beyotime Institute of Biotechnology) or 5 μM dihydroethidium (S0063; Beyotime Institute of Biotechnology) in serum-free DMEM at 37 °C for 30 min. Cells were then washed twice with PBS and immediately analyzed with a fluorescence microplate reader (ThermoFisher Scientific).

### Phagocytosis assay

Fluorescent latex beads (1 μM, L2778; Sigma-Aldrich) were used to measure the phagocytic capacity of microglia as previously described [27]. Beads were washed in PBS containing 50% FBS, centrifuged at 1000 rpm for 2 min, and then incubated with microglia in serum-free DMEM at 37 °C for 4 h. At the end of incubation, microglia were washed twice with PBS to remove the residual beads, fixed with 4% paraformaldehyde and stained with DAPI. Microglial phagocytic ability was determined using a TCS SP8 confocal laser scanning microscope with ImageJ software.

### Autophagy flux analysis

Tandem fluorescent mRFP-GFP-LC3 plasmids (Addgene, Watertown, MA) were transiently transfected into microglia according to the manufacturer’s instructions (Guangzhou RiboBio Co., Ltd, Guangzhou, China). Transfected cells were fixed with 4% paraformaldehyde and stained with DAPI after the indicated treatments. Microglial autophagy flux was imaged under a TCS SP8 confocal laser scanning microscope, and analyzed by ImageJ software to quantify RFP/GFP colocalization puncta (autophagosome), RFP puncta (autolysosome) and the autophagy flux.

### Statistical analysis

Results are presented as means ± SEM for the indicated number of experiments. Statistical analysis was performed using GraphPad Prism 8.0 (GraphPad Software, La Jolla, CA). All data were tested for normality with the Shapiro–Wilk test. Student’s *t*-test was used to compare two groups of independent samples. One-way ANOVA followed by Tukey's multiple comparisons test was used to compare three independent groups. Two-way ANOVA followed by Tukey's multiple comparisons test was used to compare multiple factors. The statistical significance threshold was set at *P* < 0.05. Detailed information on the sample size, number of replicates and statistical tests used for each experiment is provided in the figure legends.

## Results

### APOE4 impairs microglial cholesterol homeostasis in human AD brain and in a tauopathy mouse model

**Fig. 1:**
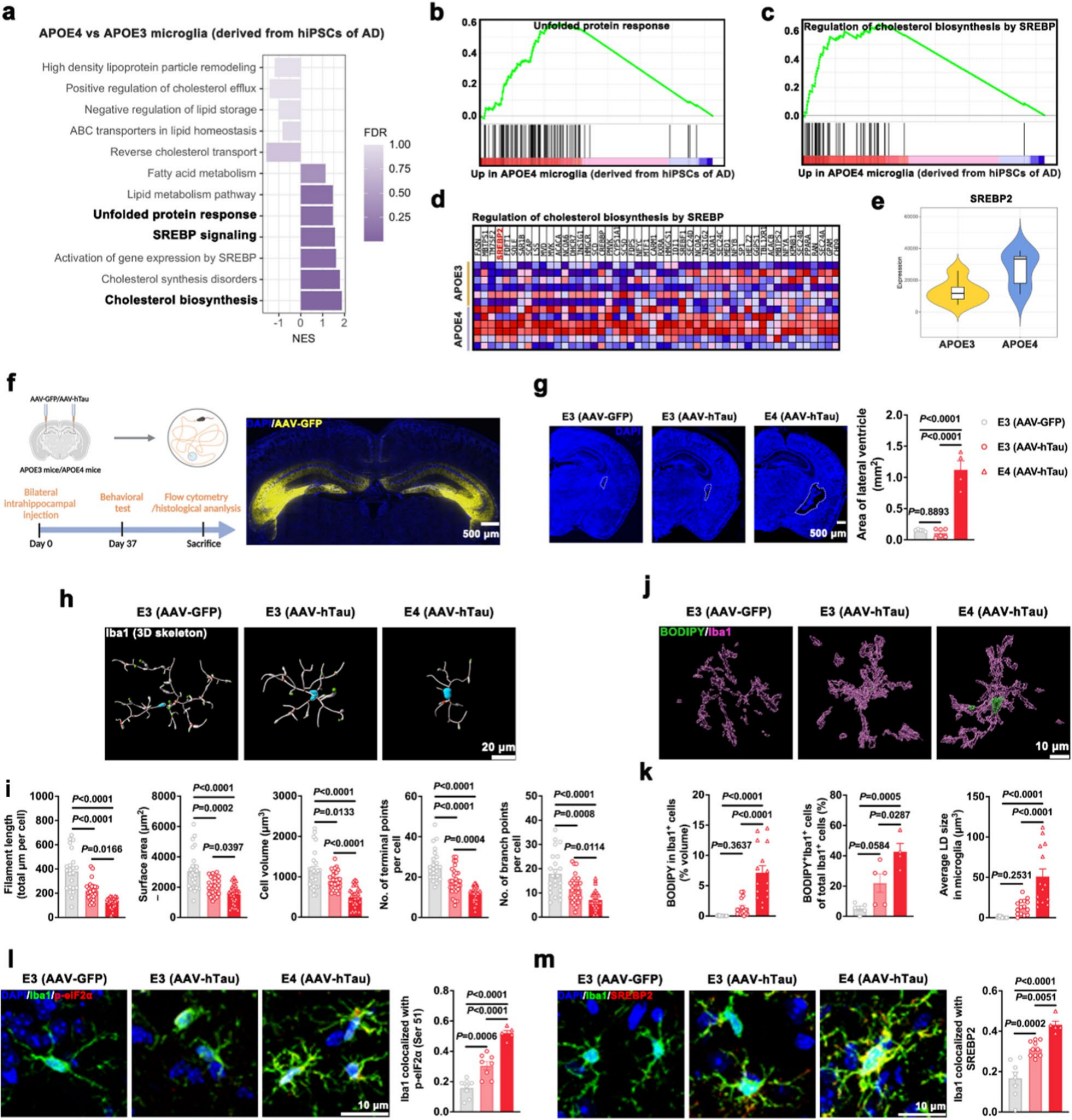
*APOE4* drives microglial cholesterol metabolic deficits in human AD and in a tauopathy mouse model. **a** Significant biological pathways and processes associated with cholesterol metabolism revealed by GSEA in *APOE4* microglia compared with *APOE3* microglia (*n* = 6) derived from hiPSCs of AD. **b**, **c** GSEA revealed enrichment of gene sets associated with unfolded protein response and regulation of cholesterol biosynthesis by SREBP pathways in *APOE4* microglia compared with *APOE3* microglia (*n* = 6) derived from hiPSCs of AD. **d** Gene expression heatmap of genes associated with the regulation of cholesterol biosynthesis by SREBP pathway in microglia. **e** The mean and variance differences for *SREBP2* expression in microglia. **f** Left, schematic of AAV-hTau injection and experimental timeline. Right, a representative image of AAV-GFP fluorescence 1 month after injection in mouse hippocampal CA3 region. Scale bar, 500 μm. **g** Mouse brain sections stained with DAPI (blue), showing the area of lateral ventricle in E3 and E4 mice after bilateral intrahippocampal injection of AAV-GFP or AAV-hTau (*n* = 4–6 fields from 3 mice in each group). Scale bar, 500 μm. **h**, **i** 3D reconstruction and relative quantification of microglia in the hippocampus of mice (*n* = 19–31 cells from 3 mice in each group). Scale bar, 20 μm. **j**, **k** 3D surface rendering and quantification of BODIPY (green) signal within Iba1^+^ microglia (purple) in the hippocampus of mice (*n* = 4 or 5 fields from 3 mice in each group). Scale bar, 10 μm. **l**, **m** Representative immunofluorescence staining and quantification of Iba1 (green) and p-eIF2α (Ser 51) (red) (**l)** or SREBP2 (red) (**m**) in the hippocampus of mice (*n* = 5–10 fields from 3 mice in each group). Scale bar, 10 μm. Nuclei were counterstained with DAPI (blue). Mean ± SEM, one-way ANOVA with Tukey’s multiple comparisons test

To gain further insights into the effects of *APOE4* on microglial cholesterol metabolism in humans, we analyzed database transcriptome data of population microglia derived from hiPSCs of AD patients with different *APOE* genotypes [4]. Differences in gene expression in *APOE4* microglia compared to *APOE3* microglia are presented through hierarchical clustering (Fig. S1a) and volcano plot (Fig. S1b). Interestingly, GSEA identified significant enrichment of gene sets associated with cholesterol biosynthesis, SREBP signaling and unfolded protein response pathways in *APOE4* microglia (Fig. 1a, b). Negatively enriched gene sets in *APOE4* microglia were associated with reverse cholesterol transport, high-density lipoprotein particle remodeling and negative regulation of lipid storage pathways (Fig. 1a and Fig. S1c, d). Regulation of cholesterol biosynthesis by the SREBP pathway was one of the most positively enriched gene sets (Fig. 1c, d). Remarkedly, *APOE4* microglia presented significantly increased expression of *SREBP2* compared to *APOE3* microglia (Fig. 1e). These results suggested that *APOE4* drives cholesterol accumulation in microglia via the SREBP2 signaling pathway during AD pathogenesis.

Given that accumulation of phosphorylated tau in the dorsal hippocampal CA3 region induces spatial learning and memory impairment in mice [28, 29], we bilaterally injected AAV-GFP or AAV-hTau encoding human full-length tau into the hippocampal CA3 region in E3 or E4 mice. The efficiency of the AAV vector was confirmed on the basis of AAV-GFP fluorescence (Fig. 1f). Compared with AAV-hTau-injected E3 mice, AAV-hTau-injected E4 mice exhibited lower activity in the open field test (Fig. S1e). In the Y maze test, AAV-hTau-injected E4 mice showed a significant reduction in spontaneous alternation behavior (Fig. S1f). In the MWM test, compared with the AAV-GFP-injected E3 mice, the AAV-hTau-injected E3 mice exhibited a significantly prolonged escape latency during the 5-day training, reduced crossings in the platform area, and less distance and time traveled in the platform quadrant after removal of the platform on day 6; these effects were more severe in AAV-hTau-injected E4 mice (Fig. S1g, h). In addition, no significant difference in the swimming velocity was observed among the three groups (Fig. S1g).

E4 mice bilaterally injected with AAV-hTau into the hippocampus exhibited severe brain atrophy accompanied by dramatic dilatation of the lateral ventricle (Fig. 1g). To explore the effect of *APOE4* on neuronal loss and tauopathy, we determined the number of NeuN^+^ cells and intensity of NeuN, MAP2, phospho-tau (p-S202/T205) (AT8) and phospho-tau (p-T231) (AT180) via immunostaining (Fig. S1i–k). Relatively fewer neuronal elements (NeuN and MAP2) and more phosphorylated tau (AT8 and AT180) were observed in the AAV-hTau-injected E4 mice.

To further assess the effect of *APOE4*-related tau pathology on microglial functional states, we performed 3D reconstruction of microglia (Fig. 1h). Microglia in the AAV-hTau-injected E3 mice had reduced process length, smaller surface area, reduced volume, and decreased terminal and branching points compared to those in the AAV-GFP-injected E3 mice; these alterations were more severe in the AAV-hTau-injected E4 mice (Fig. 1i). Large BODIPY^+^ neutral lipid inclusions were contained within microglia of AAV-hTau-injected E4 mice (Fig. 1j), and the percentage of BODIPY^+^ microglia in AAV-hTau-injected E4 mice was more than 2 folds that in AAV-hTau-injected E3 mice (Fig. 1k). Consistent with the preceding results, we observed significantly increased ER stress marker p-eIF2α (Ser 51) in Iba1^+^ microglia (Fig. 1l), as well as increased microglial SREBP2 expression and activation in the AAV-hTau-injected E4 mice compared with the E3 mice (Fig. 1m).

These observations indicated that *APOE4* aggravated activation of and cholesterol metabolic alterations in microglia, thus resulting in AD disease progression.

### *APOE4* disturbs microglial phagocytic capacity and enhances microglial MHC II-dependent antigen presentation and T cell response

**Fig. 2:**
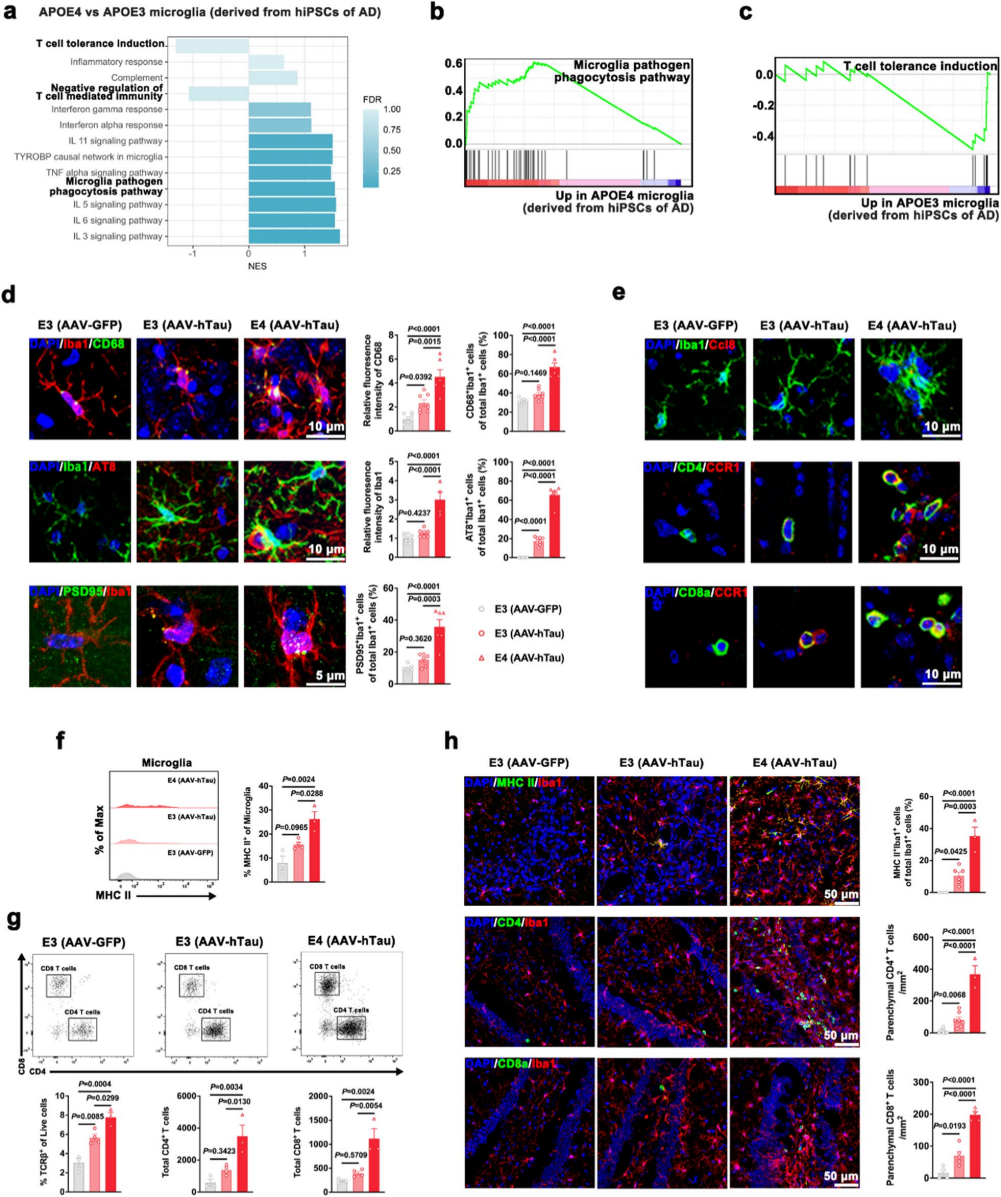
*APOE4* disturbs microglial phagocytic function and exacerbates microglia-mediated adaptive immune response. **a** Biological pathway and processes related to immune response revealed by GSEA in *APOE4* microglia compared with *APOE3* microglia derived from hiPSCs of AD (*n* = 6). **b**, **c** Microglia pathogen phagocytosis pathway and T cell tolerance induction pathway revealed by GSEA. **d** Representative images and quantification of Iba1 (red) and CD68 (green), AT8 (red) and Iba1 (green), as well as Iba1 (red) and PSD95 (green) staining in the hippocampus of mice (*n* = 4–8 fields from 3 mice in each group). **e** Representative images of Iba1 (green) and CCL8 (red), CD4 (green) and CCR1 (red), as well as CD8a (green) and CCR1 (red) staining in the hippocampus from E3 and E4 mice after bilateral intrahippocampal injection of AAV-GFP or AAV-hTau. **f** Flow cytometric gating and quantitation showing cell surface expression of MHC II in microglia of E3 and E4 mice after bilateral intrahippocampal injection of AAV-GFP or AAV-hTau (*n* = 3–4). **g** Representative flow cytometry plots demonstrating gating strategy to quantify TCRβ^+^, CD4^+^ and CD8^+^ T cells in response to AAV-hTau transduction in E3 or E4 mice (*n* = 3–4). **h** Staining and quantification of MHC II^+^ Iba1^+^ cells, CD4^+^ T cells and CD8a^+^ cells in the hippocampus of E3 and E4 mice after bilateral intrahippocampal injection of AAV-GFP or AAV-hTau (*n* = 3–11 fields from 3 mice in each group). Nuclei were counterstained with DAPI (blue). Mean ± SEM, one-way ANOVA with Tukey’s multiple comparisons test

GSEA analysis further revealed specific and significant enrichment of gene sets associated with microglial pathogen phagocytosis pathway and inflammatory response. The negatively enriched gene sets in *APOE4* microglia compared to *APOE3* microglia included negative regulation of T cell-mediated immunity and T cell tolerance induction (Fig. 2a–c).

AAV-hTau-injected E4 mice showed elevated hippocampal expression of CD68 and Iba1 and increased number of CD68^+^ microglia (Fig. 2d and S1l), indicating that *APOE4* affects the hTau-induced activation and the phagocytic capacity of microglia. Next, we measured microglial engulfment of extracellular tau and synaptic elements by quantifying AT8^+^ Iba1^+^ cells and PSD95^+^ Iba1^+^ cells. Significantly more AT8 puncta and PSD95 were found in microglia in the hippocampus of AAV-hTau-injected E4 mice compared to AAV-hTau-injected E3 mice (Fig. 2d). These data together suggested that *APOE4* is the driving force of tau pathogenesis, predominantly through regulation of the microglial phagocytic capacity.

The chemotactic response of microglia-derived chemokines in mediating the infiltration of T cells has been reported in neurological diseases [2, 30]. Compared with AAV-hTau-injected E3 mice, microglia expressing CCL8, and CD4 or CD8 T cells expressing CCR1 were detected in the hippocampus of AAV-hTau-injected E4 mice (Fig. 2e).

Flow cytometric stratification and quantification revealed increased MHC II expression on resident microglia in the hippocampus of AAV-hTau-injected E4 mice (Fig. 2f, Fig. S2a). Flow cytometric analysis further showed an expansion of the T cell compartment in the AAV-hTau-injected E3 mice compared with the AAV-GFP-injected E3 mice, and a even higher number of infiltrating T cells, primarily CD4 T cells, in the brains of AAV-hTau-injected E4 mice (Fig. 2g). Significant upregulation of MHC II protein was observed in Iba1^+^ microglia in AAV-hTau-injected E4 mice compared to AAV-hTau-injected E3 mice (Fig. 2h). Immunostaining of the hippocampus showed prominent interactions between Iba1^+^ microglia and CD4 T cells or CD8 T cells (Fig. 2h). These observations indicated that *APOE4* influences the phagocytic capacity and antigen presentation by microglia, thus resulting in the priming of the adaptive immune response in the tauopathy mouse model.

### TRPV1 agonist capsaicin reduces cholesterol packing in microglia in APOE4-related tauopathy through increasing uptake of exogenous Ca^2+^

**Fig. 3:**
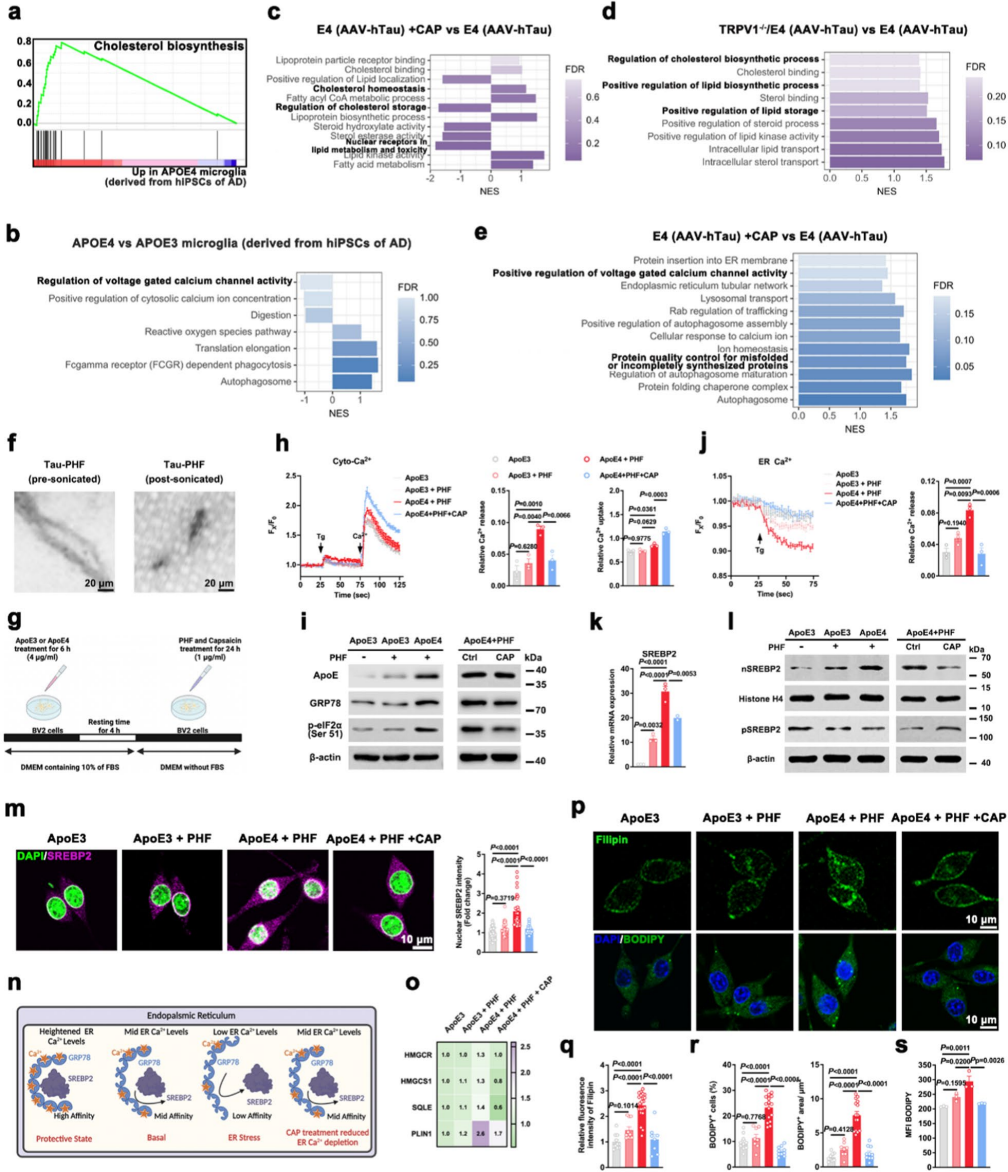
TRPV1 agonist blocks SREBP2 activation and cholesterol accumulation in ApoE4 + PHF microglia. **a** GSEA revealed enrichment of the cholesterol biosynthesis pathway in *APOE4* microglia compared with *APOE3* microglia derived from hiPSCs of AD. **b** Biological pathways and processes associated with voltage-gated calcium channel revealed by GSEA in *APOE4* microglia compared with *APOE3* microglia derived from hiPSCs of AD (*n* = 6). **c**–**e** Biological pathways and processes associated with cholesterol metabolism (**c, d**) and voltage-gated calcium channel (**e**) revealed by GSEA in microglia isolated from the hippocampus and cortex of 4-month-old mice (*n* = 3). **f** Representative TEM images of pre-sonicated PHF and post-sonicated PHF. **g** Experimental procedure for lipid packing in BV2 cells stimulated with PHF. **h** Thapsigargin-induced ER Ca^2+^ release and Ca^2+^ uptake detected using a Ca^2+^ sensitive dye Fluo-4 AM in Ca^2+^-free buffer. The traces and quantitative results show relative fluorescence intensity of intracellular Ca^2+^ (*n* = 3, biological replicates). **i** Immunoblots of ApoE, GRP78 and p-eIF2α (Ser 51). **j** Dynamic changes of ER Ca^2+^ labeled with the low-affinity Ca^2+^-sensitive dye Mag-fluo-4 AM in BV2 cells after thapsigargin stimulation in a Ca^2+^-free extracellular solution (*n* = 3, biological replicates). **k** Relative mRNA expression of *SREBP2* (*n* = 3, biological replicates). **l** Immunoblots of pre-mature SREBP2 and the activated nuclear SREBP2. **m** Representative immunofluorescent images and quantitative analysis of the cellular localization of SREBP2 (purple) (*n* = 16 to 36 cells from 3 biological replicates). Nuclei were counterstained with DAPI (green). **n** A model of how Ca^2+^ promotes the sequestration of SREBP2 in the ER. **o** Heatmap of the relative mRNA expression of *HMGCR*, *HMGCS1*, *SQLE* and *PLIN1* (*n* = 3, biological replicates). **p**–**r** Representative fluorescent images and quantification of filipin and BODIPY staining (*n* = 8–21 fields from 3 biological replicates). Nuclei were counterstained with DAPI (blue). Scale bar, 10 μm. **s** Mean fluorescence intensity (MFI) of BODIPY quantified with flow cytometry in BV2 cells (*n* = 3, biological replicates). Mean ± SEM, one-way ANOVA with Tukey’s multiple comparisons test

Cholesterol biosynthesis was one of the most positively enriched gene sets (Fig. 3a). Further analysis of these data revealed that regulation of voltage-gated calcium channel activity and positive regulation of cytosolic calcium ion concentration were downregulated in *APOE4* microglia compared with *APOE3* microglia derived from hiPSCs of AD patients (Fig. 3b). TRPV1 is a voltage-gated nonselective cation channel with Ca^2+^ permeability. Prior studies have established involvement of microglial TRPV1 in the role of microglia in *APOE4*-dependent neurodegeneration [2, 3, 5, 31]. We therefore examined cholesterol metabolism of microglia in E4 (AAV-hTau) mice, E4 (AAV-hTau) + CAP mice with capsaicin treatment for 1 month, or TRPV1^−/−^/E4 (AAV-hTau) mice lacking the microglial *TRPV1* gene. GSEA identified significant enrichment of gene sets of lipoprotein biosynthetic process and cholesterol homeostasis in the microglia of E4 (AAV-hTau) + CAP mice compared with E4 (AAV-hTau) mice (Fig. 3c). Negatively enriched gene sets included nuclear receptors in lipid metabolism and toxicity and regulation of cholesterol storage (Fig. 3c). Microglia of TRPV1^−/−^/E4 (AAV-hTau) mice showed significant enrichment of gene sets associated with intracellular lipid transport, positive regulation of lipid storage, positive regulation of lipid biosynthetic process, and regulation of cholesterol biosynthetic process compared with E4 (AAV-hTau) mice (Fig. 3d). GSEA analysis about cellular homeostasis further suggested that gene sets for positive regulation of voltage-gated calcium channel activity, cellular response to calcium ion and protein quality control for misfolded or incompletely synthesized proteins, were upregulated in microglia of E4 (AAV-hTau) mice after treatment with capsaicin for 1 month (Fig. 3e).

Recombinant human tau protein was used to prepare fibrils, as verified by TEM [32] (Fig. 3f). To discover whether the presence of human ApoE isoforms might affect microglial function in the setting of tauopathy, we added 4 μg/ml purified ApoE3 or ApoE4 protein to cultured BV2 cells for 6 h with 10% FBS, to ensure cholesterol loading. Then, the cells were incubated with fresh medium for 4 h and exposed to 1 μg/ml PHF for 24 h after exposure to vehicle or capsaicin [24, 25] (Fig. 3g).

Given that TRPV1 functions as a Ca^2+^ channel, Ca^2+^-sensitive dye Fluo-4 AM was used to measure changes in intracellular Ca^2+^. Microglia treated with ApoE4 in the presence of PHF showed significant increase of Tg-induced ER Ca^2+^ release and slight exogenous Ca^2+^ uptake (CaCl_2_) into the cytoplasm than the cells treated with ApoE3 in the presence or absence of PHF. In addition, ApoE4 + PHF microglia treated with capsaicin significantly diminished ER Ca^2+^ release and enhanced exogenous Ca^2+^ uptake (Fig. 3h). These data indicate that *APOE4*-related tauopathy results in disturbance of microglial calcium homeostasis, and inhibition of the disturbance through TRPV1 rescues the cytosolic calcium ion concentration.

Consistent with previous reports that ApoE4 is involved in ER stress and calcium signaling in AD pathogenesis [33–35], intracellular accumulation of ApoE protein and expression of the ER stress markers glucose regulated proteins (GRP78) and p-eIF2α (Ser 51) were significantly upregulated in microglia treated with ApoE4 in the presence of PHF than in the cells treated with ApoE3 in the presence or absence of PHF. Treatment with capsaicin decreased ApoE accumulation and ER stress in the ApoE4 + PHF microglia (Fig. 3i and S3a). The low-affinity Ca^2+^-sensitive dye Mag-fluo-4 AM was used to examine the dynamic changes of ER Ca^2+^ in microglia. Results showed a significant decrease in Tg-induced ER Ca^2+^ release in ApoE4 + PHF microglia after capsaicin treatment (Fig. 3j). A low level of ER Ca^2+^ triggers the transcriptional activation of SREBP2, a main transcriptional regulator of cholesterol metabolism and homeostasis [36, 37]. Consistent with that, the enhanced capacity of ER Ca^2+^ store after capsaicin treatment decreased mRNA expression of *SREBP2* in ApoE4 + PHF microglia (Fig. 3k). In addition, treatment with capsaicin decreased SREBP2 re-localization from the perinuclear region to the nucleus (an activated isoform) in ApoE4 + PHF microglia (Figs. 3l, m and S3b). Collectively, these data suggest that the TRPV1 agonist capsaicin reduced ER stress, reversed depletion of ER Ca^2+^ and decreased nuclear localization of SREBP2 in ApoE4 + PHF microglia (Fig. 3n).

SREBP2 is main transcription factor regulating cholesterol biosynthesis, uptake and transport [38, 39]. The mRNA expression of cholesterol-biosynthesis genes regulated by SREBP2 (*HMGCR*, *HMGCS1*, *SQLE* and *PLIN1*) was downregulated in ApoE4 + PHF microglia after pretreatment with 1 μM capsaicin (Figs. 3o and S3c). Furthermore, capsaicin treatment increased protein levels of cholesterol efflux transporters ABCA1 and ABCG1 in ApoE4 + PHF microglia (Fig. S3d, e). Next, we examined mechanisms of ABCA1 intracellular trafficking. TRPV1 agonist capsaicin increased ABCA1 colocalization with the recycling endosome marker Rab11 and decreased its colocalization with the late endosome marker LAMP2 (lysosomal associated membrane protein 2) in microglia treated with PHF (Fig. S3f). The accumulation of cholesterol was further confirmed by staining with filipin, a cholesterol-binding fluorescent macrolide. Fluorescence images showed that the TRPV1 agonist capsaicin diminished intracellular filipin staining in ApoE4 + PHF microglia (Fig. 3p, q). The disrupted cholesterol homeostasis promoted lipid droplet formation. Fluorescence staining (Fig. 3p, r) and flow cytometry (Fig. 3s) revealed decreases in the BODIPY^+^ area, the number of BODIPY^+^ cells and the BODIPY mean fluorescence in ApoE4 + PHF microglia after pretreatment with capsaicin. These data supported that TRPV1, by regulating cytosolic Ca^2+^ concentration, plays a major role in regulating cholesterol metabolism in microglia in the context of *APOE4*-related pathology.

### TRPV1 agonist capsaicin reduces MHC II-dependent antigen presentation and neuroinflammation of microglia in the context of *APOE4*-related tauopathy

**Fig. 4:**
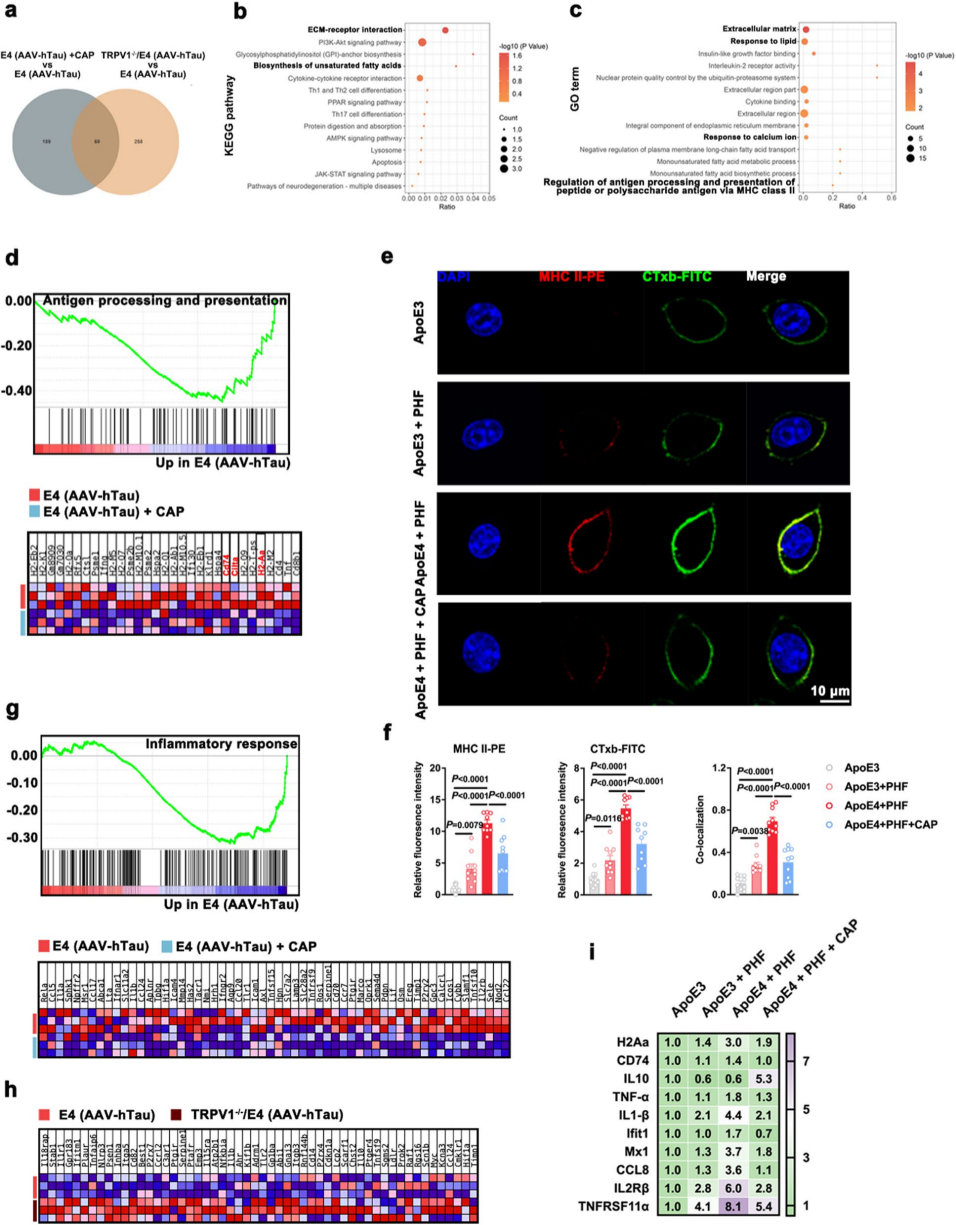
TRPV1 agonist alleviates the MHC II-dependent antigen presentation and inflammatory response in the ApoE4 + PHF microglia. **a** Venn diagram depicting the number of genes that were significantly altered between E4 (AAV-hTau) + CAP *vs* E4 (AAV-hTau) and between TRPV1^−/−^/E4 (AAV-hTau) *vs* E4 (AAV-hTau) (*n* = 3). **b, c** Bubble plots of KEGG pathway (**b**) and GO pathway analysis (**c**) from 69 transcripts that were significantly altered in both comparisons in **a**. **d** GSEA and gene expression heatmap of gene set associated with antigen processing and presentation in microglia isolated from the hippocampus and cortex of 4-month-old mice (*n* = 3). **e**, **f** Representative immunofluorescence images and quantification for MHC II-PE (red) and CTxb-FITC (green) in ApoE4 + PHF BV2 cells after exposure to vehicle or capsaicin (CAP) (*n* = 9–10 fields from 3 biological replicates). **g** GSEA and gene expression heatmap of gene set associated with inflammatory response in microglia isolated from the hippocampus and cortex of 4-month-old E4 (AAV-hTau) and E4 (AAV-hTau) + CAP mice (*n* = 3). **h** Gene expression heatmap of inflammatory response in microglia isolated from the hippocampus and cortex of E4 (AAV-hTau) and 4-month-old TRPV1^−/−^/E4 (AAV-hTau) mice (*n* = 3 samples). **i** Heatmap of the relative mRNA expression of *H2Aa*, *CD74*, *IL10*, *TNF-α*, *IL-1β*, *Ifit1*, *Mx1*, *CCL8*, *IL2Rβ* and *TNFRSF11α* (*n* = 3 biological replicates). Mean ± SEM, one-way ANOVA with Tukey’s multiple comparisons test

Extensive efforts have elucidated a crucial role of microglial immunoreactivity in driving *APOE4*-related tauopathy and neurodegeneration [2–4]. To test whether the TRPV1 channel affects microglial immunoreactivity, we compared microglial transcriptional profiles based on scRNA-seq data sets between E4 (AAV-hTau) + CAP mice and E4 (AAV-hTau) mice as well as between TRPV1^−/−^/E4 (AAV-hTau) mice and E4 (AAV-hTau) mice, and identified 69 common differentially expressed genes between the data sets (Fig. 4a). Kyoto Encyclopedia of Genes and Genomes (KEGG) enrichment analysis of the 69 genes revealed enrichment of KEGG pathways including extracellular matrix (ECM)-receptor interaction, biosynthesis of unsaturated fatty acids, cytokine-cytokine receptor interaction, and lysosomes (Fig. 4b). Gene Ontology (GO) enrichment analysis revealed significant enrichment terms including ECM, response to lipid, response to calcium ion, and regulation of antigen processing and presentation of peptide or polysaccharide antigen via MHC class II (Fig. 4c).

Antigen processing and presentation was one of the most negatively enriched gene sets in microglia of E4 (AAV-hTau) + CAP mice compared with E4 (AAV-hTau) mice. Remarkedly, microglia of E4 (AAV-hTau) + CAP mice presented significantly decreased expression of *CD74*, *H2-Aa* and *Ciita* (Fig. 4d). We further investigated whether increased cholesterol content might lead to an increase in lipid rafts, thus affecting the distribution of the MHC II receptor that is associated with antigen presentation and is known to localize in lipid rafts [40]. ApoE4 increased the colocalization of MHC II with CTxb (a marker of lipid rafts) in microglia treated with PHF compared with cells treated with ApoE3 in the presence or absence of PHF. Treatment with capsaicin significantly decreased MHC II in lipid rafts at the cell membranes of ApoE4 + PHF microglia (Fig. 4e, f).

GSEA analysis identified a negatively enriched gene set of inflammatory response in microglia from E4 (AAV-hTau) + CAP mice compared with E4 (AAV-hTau) mice (Fig. 4g). Conversely, microglia in TRPV1^−/−^/E4 (AAV-hTau) mice presented significantly increased expression of genes associated with inflammation compared with microglia in E4 (AAV-hTau) mice (Fig. 4h). In addition, ApoE4 + PHF microglia showed higher mRNA expression of antigen presentation-related genes (*H2Aa* and *CD74*) and pro-inflammatory genes (*IL10*, *TNF-α*, *IL-1β*, *Ifit1*, *Mx1*, *CCL8*, *IL2Rβ* and *TNFRSF11α*) than microglia treated with ApoE3 in the presence or absence of PHF, and activation of TRPV1 diminished the expression of antigen presentation-related genes and pro-inflammatory genes (Figs. 4i and S3g, h).

Together, these findings validated the in vivo relevance of the mechanism identified in vitro and confirmed that TRPV1 regulates microglial cholesterol metabolism and immune function, thus suggesting potential therapeutic targets to prevent *APOE4*-related immune response in tauopathies.

### TRPV1 agonist enhances autophagic flux and reduces lysosomal cholesterol content in microglia in the context of *APOE4*-related tauopathy

**Fig. 5:**
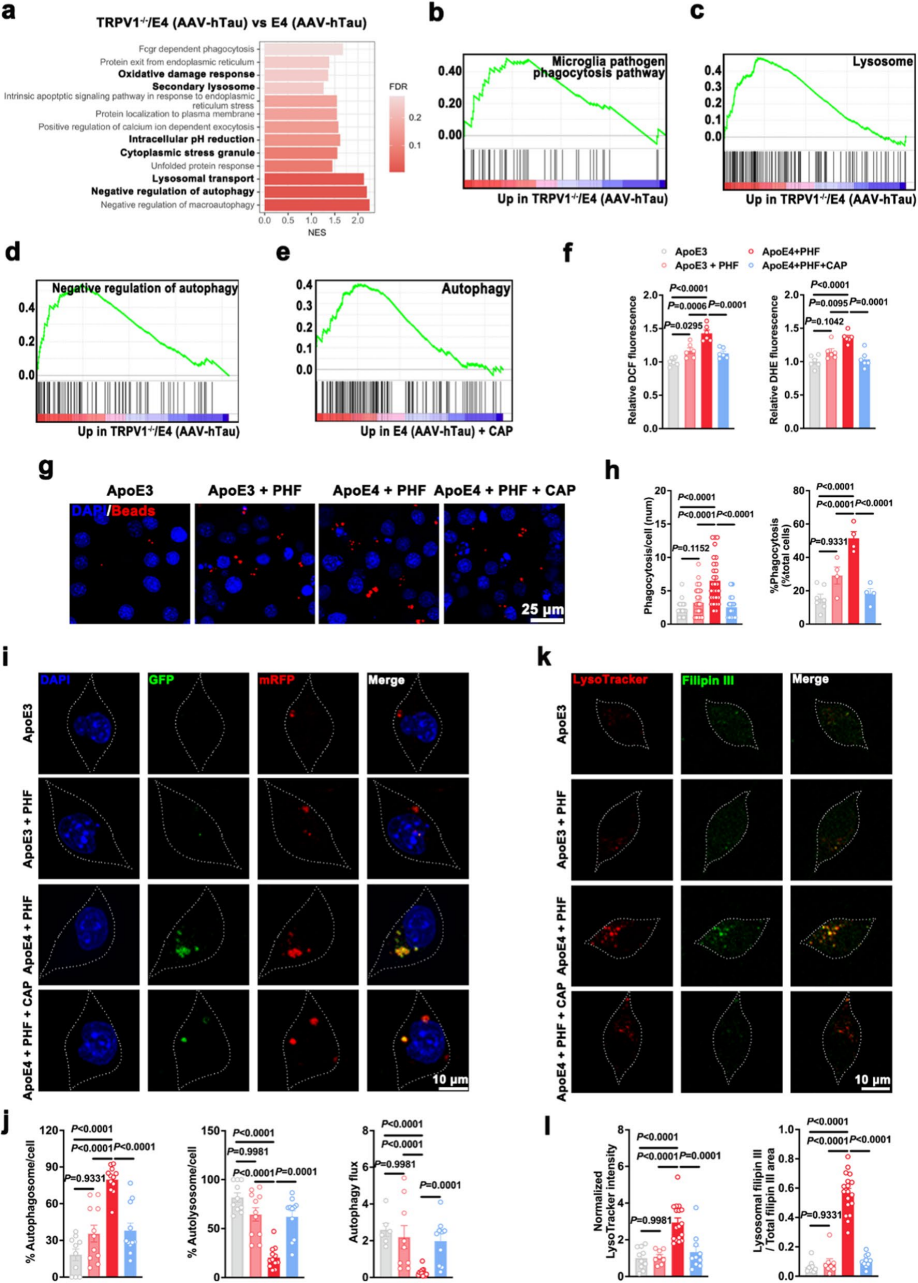
TRPV1 agonist promotes autophagic activity and reduces lysosomal cholesterol accumulation in ApoE4 + PHF microglia. **a** Biological pathways associated with cell-degradative process revealed by GSEA in microglia isolated from the hippocampus and cortex of 4-month-old mice (*n* = 3). **b-d** GSEA revealed enrichment of gene sets associated with microglial pathogen phagocytosis pathway (**b**), lysosome (**c**) and negative regulation of autophagy (**d**) in microglia isolated from the hippocampus and cortex of 4-month-old TRPV1^−/−^/E4 (AAV-hTau) mice (*n* = 3). **e** GSEA revealed enrichment of gene set associated with autophagy in microglia isolated from the hippocampus and cortex of 4-month-old E4 (AAV-hTau) + CAP mice (*n* = 3). **f** Intracellular total ROS content and superoxide level in ApoE4 + PHF BV2 cells after exposure to vehicle or capsaicin (*n* = 6 biological replicates). **g**, **h** Representative images (**g**) and analysis (**h**) of phagocytic activity in the fluorescent beads uptake assay (*n* = 4–7 fields from 3 biological replicates). **i**, **j** Representative images (**i**) and analysis (**j**) of autophagic activity determined by mRFP-GFP-LC3 plasmid (*n* = 10–12 fields from 3 biological replicates). Nuclei were counterstained with DAPI (blue). **k**, **l**. Representative images (**k**) and quantification (**l**) of LysoTracker and filipin III staining in ApoE4 + PHF BV2 cells after exposure to vehicle or capsaicin (*n* = 8–18 fields from 3 biological replicates). Mean ± SEM, one-way ANOVA with Tukey’s multiple comparisons test

We next asked whether altered cholesterol content affects the relative activity of cellular degradation pathways in microglia. GSEA analysis revealed significant enrichment of gene sets for microglia pathogen phagocytosis pathway, negative regulation of autophagy, lysosome, intracellular pH reduction, and oxidative damage response in the microglia of TRPV1^−/−^/E4 (AAV-hTau) mice compared to E4 (AAV-hTau) mice (Fig. 5a–d). In contrast, microglia presented significant enrichment of autophagy in microglia from the E4 (AAV-hTau) + CAP mice (Fig. 5e).

To determine the oxidative-stress status of microglia in response to *APOE4*-related tauopathy, we assessed ROS level and superoxide anion production. ApoE4 upregulated the levels of ROS and superoxide anion radical in microglia treated with PHF compared to ApoE3. Treatment with capsaicin significantly diminished the oxidative-stress status of ApoE4 + PHF microglia (Fig. 5f). Similar to our previous analysis, the phagocytic capacity was significantly increased in microglia treated with ApoE4 in the presence of PHF, but capsaicin decreased the phagocytic ability (Fig. 5g, h).

As free cholesterol accumulation in lysosomes has been found to interfere with lysosomal functions, resulting in impairment of the autophagy lysosomal pathway [41], we assessed autophagic activity and cholesterol accumulation in the lysosomes of microglia in response to *APOE4*-related tauopathy. mRFP-GFP-tagged LC3 reporter was expressed in microglia to visually analyze the altered autophagic flux. We detected increased autophagosomes (remaining yellow dots in Fig. 5i) relative to autolysosomes in microglia treated with ApoE4 in the presence of PHF, indicating impaired autophagy lysosomal pathway. Treatment with TRPV1 agonist capsaicin restored the autophagy lysosomal pathway in microglia (Fig. 5i, j). We then assessed cholesterol content in the lysosomes of microglia using a cholesterol-binding fluorescent macrolide filipin along with LysoTracker dye to label lysosomes. ApoE4 significantly increased the accumulation of cholesterol in lysosomes of microglia treated with PHF compared to ApoE3, while treatment with capsaicin significantly decreased it (Fig. 5k, l). In summary, these data indicate that TRPV1 agonist promotes autophagic activity and reduces cholesterol accumulation in lysosomes of microglia in response to *APOE4*-related pathology, and subsequently maintains the cellular homeostasis.

### Pharmacological activation of TRPV1 rescues memory deficits and decreases microglial lipid accumulation and T cell infiltration in E4 mice with tauopathy

**Fig. 6:**
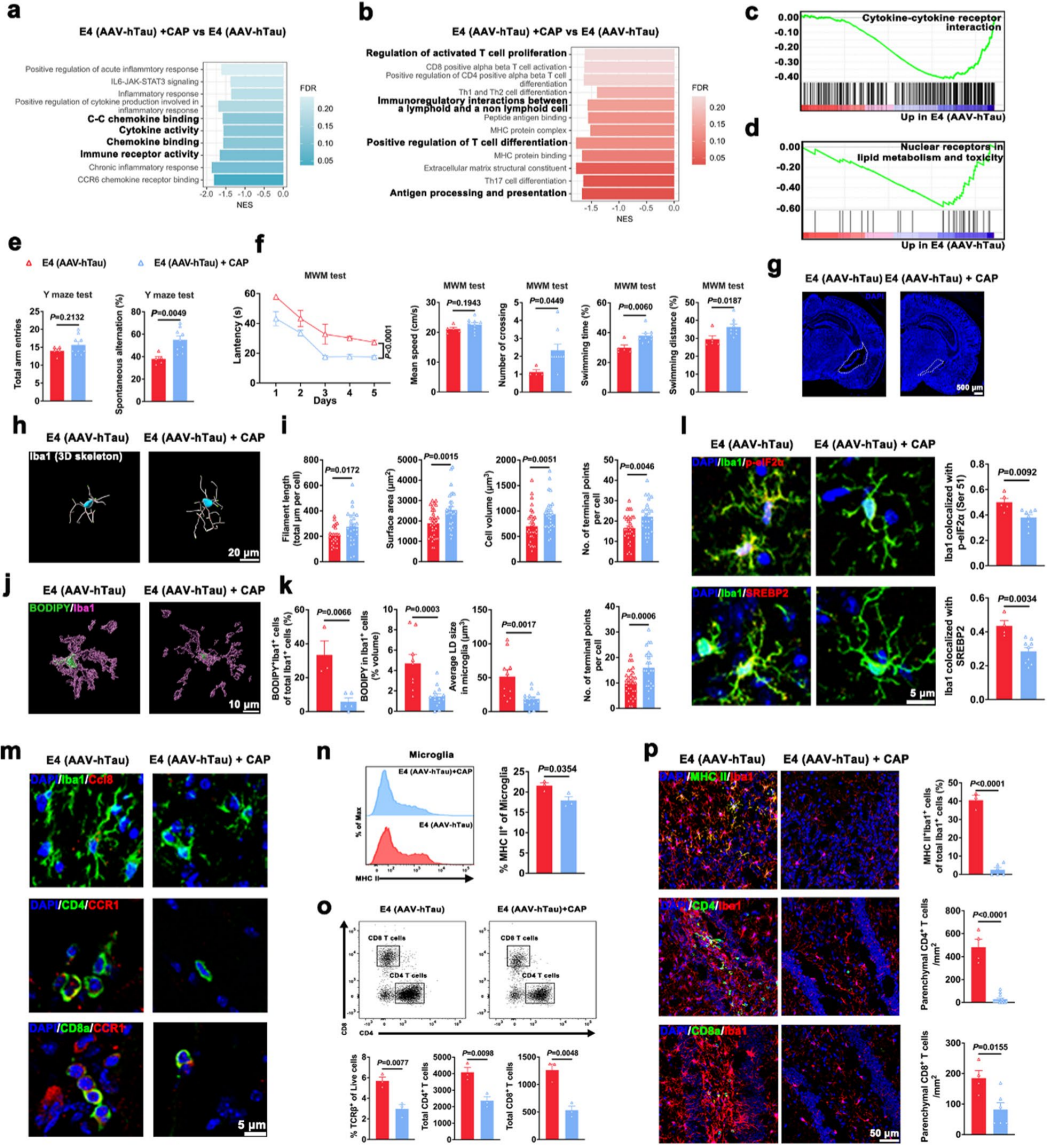
TRPV1 agonist capsaicin ameliorates neurodegeneration, microglial lipid accumulation and T cell infiltration in E4 mice with tauopathy. **a–d** Biological pathways and processes related to chemotaxis (**a**), microglia-mediated T cell response (**b**), cytokine and cytokine receptor interaction (**c**), and nuclear receptors in lipid metabolism and toxicity (**d**) revealed by GSEA in microglia isolated from the hippocampus and cortex of 4-month-old mice (*n* = 3). **e** Total arm entries and spontaneous alternation in the Y maze test. *n* = 5 and 9 mice for E4 (AAV-hTau) and E4 (AAV-hTau) + CAP, respectively. **f** MWM test showed significant differences in escape latency during training period, number of crossings, percentage of distance travelled and time spent in platform quadrant during probing period between E4 (AAV-hTau) mice (*n* = 5) and E4 (AAV-hTau) + CAP mice (*n* = 9). **g** Representative images of the area of lateral ventricle. Nuclei were counterstained with DAPI (blue). **h**, **i** 3D reconstruction and relative quantification of microglia (*n* = 23–33 cells from 3 mice in each group). Scale bar, 20 μm. **j**, **k** Representative 3D surface rendering and quantification of BODIPY (green) signal within Iba1^+^ microglia (purple) (*n* = 3–5 fields from 3 mice in each group). **l** Representative images and quantification of Iba1 co-localization with p-eIF2α (Ser 51) or SREBP2 (*n* = 4–9 fields from 3 mice in each group). **m** Representative fluorescence images for Iba1 (green) and CCL8 (red), CD4 (green) and CCR1 (red), as well as CD8a (green) and CCR1 (red). **n**, **o** Flow cytometric gating and quantitation showing MHC II cell surface expression in CNS microglia (**n**) and gating strategy to quantify TCRβ^+^, CD4^+^ and CD8^+^ T cells (**o**) (*n* = 3). **p** Representative images and quantification of MHC II^+^ Iba1^+^ cells, CD4^+^ T cells and CD8a^+^ T cells (*n* = 3–14 fields from 3 mice in each group). Nuclei were counterstained with DAPI (blue). Mean ± SEM, unpaired *t*-test

After capsaicin treatment for 1 month (Fig. S4a), 66 upregulated genes and 192 downregulated genes were detected in microglia from hippocampus and cortex of E4 (AAV-hTau) mice compared to those without capsaicin treatment (Fig. S4b, c). The BPs enriched in the 66 upregulated genes included voltage-gated calcium channel activity involved in regulation of cytosolic calcium levels, cellular response to calcium ions, and protein localization to ER (Fig. S4d). The BPs enriched in the 129 downregulated genes included extracellular matrix, T cell meandering migration, antigen processing and presentation, and myeloid cell activation involved in the immune response (Fig. S4e). GSEA analysis revealed enrichment of downregulated gene sets involved in chronic inflammatory response, chemokine binding, cytokine activity, ECM structural constituent, cytokine-cytokine receptor interaction, and nuclear receptors in lipid metabolism and toxicity in microglia of E4 (AAV-hTau) mice after treatment with capsaicin for 1 month (Figs. 6a–d and S4f). Microglia from E4 (AAV-hTau) + CAP mice also had significantly negatively enriched gene sets for antigen processing and presentation, positive regulation of T cell differentiation, immunoregulatory interactions between a lymphoid and a non-lymphoid cell, and regulation of activated T cell proliferation, compared with E4 (AAV-hTau) mice (Fig. 6b).

Consistent with the positively enriched gene set of long-term potentiation in E4 (AAV-hTau) + CAP mice (Fig. S5a), capsaicin treatment reversed the spatial recognition memory in AAV-hTau-injected E4 mice in Y maze test (Fig. 6e). No significant difference was observed in the performance in open field test between E4 (AAV-hTau) mice and E4 (AAV-hTau) + CAP mice (Fig. S5b). Moreover, the E4 (AAV-hTau) + CAP mice showed significantly shortened escape latency, increased number of crossings, as well as increased distance and time traveled in the platform quadrant in MWM test. Moreover, the two groups showed no difference in the swimming velocity (Figs. 6f and S5c). Immunostaining revealed markedly reduced lateral ventricle area (Figs. 6g and S5d), neuronal loss (Fig. S5e) and tauopathy (Fig. S5f) in E4 (AAV-hTau) + CAP mice.

Microglia exhibited increased process length, surface area, volume, as well as terminal and branch points in E4 (AAV-hTau) + CAP mice (Fig. 6h, i). Consistent with the GSEA data (Fig. 6d), few BODIPY^+^ neutral lipid inclusions were contained within microglia of E4 (AAV-hTau) + CAP mice (Fig. 6j). The percentage of BODIPY^+^ microglia was more than six-fold higher in the E4 (AAV-hTau) mice compared with E4 (AAV-hTau) + CAP mice (Fig. 6k). Capsaicin treatment decreased the levels of ER stress marker p-eIF2α (Ser 51) and SREBP2 activation in hippocampal microglia (Fig. 6l).

Consistent with the decreased microglial engulfment of extracellular tau and synaptic elements, capsaicin treatment decreased the phagocytic capacity of hippocampal microglia (Fig. S5g, h). These observations indicated that TRPV1 regulates the immune state and cholesterol metabolism of microglia in AAV-hTau-injected E4 mice.

The expression of CCL8 in microglia, CCR1 in CD4 cells, or CD8 in T cells were decreased in the hippocampus of E4 (AAV-hTau) + CAP mice (Fig. 6m), which was consistent with the significant negatively enriched gene set identified by GSEA (Fig. 6a, c). The E4 (AAV-hTau) + CAP mice showed decreased MHC II expression on resident microglia (Fig. 6n). Flow cytometric analysis showed a smaller T cell fraction in E4 (AAV-hTau) + CAP mice than in E4 (AAV-hTau) mice (Fig. 6o). Immunostaining showed rare microglial MHC II expression, T cell infiltration and interactions between microglia and CD4 T cells or CD8 T cells in the hippocampus of E4 (AAV-hTau) + CAP mice (Fig. 6p). Together, these results suggest associations of TRPV1 activity with microglia-dependent antigen presentation and the adaptive immune response in E4 mice with tauopathy.

### Microglial-specific knockout of TRPV1 exacerbates neurodegeneration, microglial cholesterol accumulation and microglia-primed adaptive immunity in E4 mice with tauopathy

**Fig. 7:**
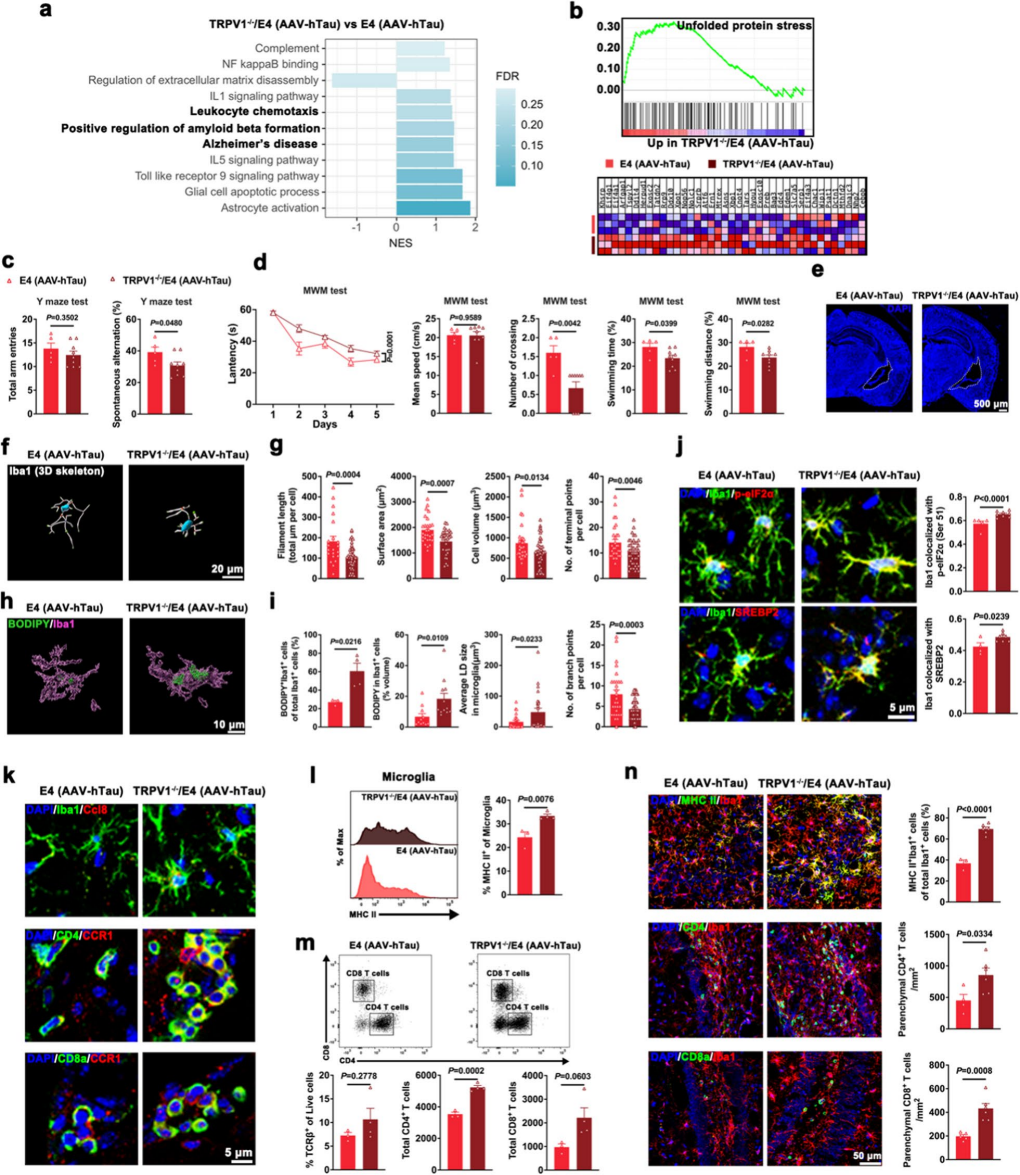
Microglia-specific TRPV1 deficiency exacerbates memory impairments, microglial lipid accumulation and microglia-mediated adaptive immunity in E4 mice with tauopathy. **a** Biological pathways and processes associated with leukocyte chemotaxis and Alzheimer’s disease revealed by GSEA in microglia isolated from the hippocampus and cortex of 4-month-old mice (*n* = 3). **b** GSEA-revealed enrichment and gene expression heatmap of gene set associated with unfolded protein response in microglia isolated from the hippocampus and cortex of 4-month-old mice (*n* = 3). **c** Total arm entries (*n* = 5 and 9 mice for E4 (AAV-hTau) and TRPV1^−/−^/E4 (AAV-hTau), respectively) and spontaneous alternation (*n* = 4 and 5, rspectively) in the Y maze test. **d** MWM test of age-matched E4 (AAV-hTau) mice (*n* = 5) and TRPV1^−/−^/E4 (AAV-hTau) mice (*n* = 9). **e** Representative images of the area of lateral ventricle. Nuclei were counterstained with DAPI (blue). **f**, **g** Representative 3D reconstruction and relative quantification of microglia. *n* = 21–46 cells from 3 mice in each group. **h**, **i** Representative 3D surface rendering and quantification of BODIPY (green) signal within Iba1^+^ microglia (purple) (*n* = 3 or 4 fields from 3 mice in each group). Scale bar, 10 μm. **j** Representative images and quantification of Iba1 co-localization with p-eIF2α (Ser 51) and SREBP2 (*n* = 4–9 fields from 3 mice in each group). **k** Representative fluorescence images for Iba1 (green) and CCL8 (red), CD4 (green) and CCR1 (red), as well as CD8a (green) and CCR1 (red) in the hippocampus. **l**, **m** Flow cytometric gating and quantitation showing MHC II cell surface expression in CNS microglia (**l**) and gating strategy to quantify TCRβ^+^, CD4^+^ and CD8^+^ T cells (**m**). *n* = 3 and 4 mice for E4 (AAV-hTau) and TRPV1^−/−^/E4 (AAV-hTau), respectively. **n** Representative images and quantification of MHC II^+^ Iba1^+^ cells, CD4^+^ T cells and CD8^+^ T cells (*n* = 3–6 fields from 3 mice in each group). Nuclei were counterstained with DAPI (blue). Mean ± SEM, unpaired *t*-test

We further examined the role of microglial TRPV1 in tau pathology by bilaterally injecting AAV-hTau into the hippocampal CA3 region of TRPV1^−/−^/E4 mice (Fig. S6a). Microglia isolated from the cortex and hippocampus of TRPV1^−/−^/E4 (AAV-hTau) mice were subjected to scRNA-seq (Fig. S6b). We identified 116 upregulated and 211 downregulated genes in microglia of TRPV1^−/−^/E4 (AAV-hTau) mice compared to E4 (AAV-hTau) mice (Fig. S6b, c). BPs enriched in the 116 upregulated genes were associated with complement activation and alternative pathway, response to interleukin, chemokine ligand production, inflammatory response, ECM organization, transcription factor activity, and negative regulation of low-density lipoprotein particle clearance (Fig. S6d). BPs enriched in the 211 downregulated genes were associated with negative regulation of CD4 biosynthetic process, apoptotic cell clearance, regulation of innate immune response, negative regulation of antigen processing and presentation of peptide via MHC II, fatty acid metabolic process, and regulation of calcium ion binding (Fig. S6e). GSEA analysis showed significant enrichment of gene sets involved in Alzheimer’s disease, positive regulation of amyloid-β formation, leukocyte chemotaxis, unfolded protein stress and linoleic acid metabolism in microglia of TRPV1^−/−^/E4 (AAV-hTau) mice (Figs. 7a, b and S6f).

Compared with AAV-hTau-injected E4 mice, TRPV1^−/−^/E4 (AAV-hTau) mice exhibited significantly lower activity in the open field tests (Fig. S7a) and impaired spatial recognition memory in the Y maze test (Fig. 7c). The TRPV1^−/−^/E4 (AAV-hTau) mice also showed longer escape latency, decreased number of crossings, shorter distance and less time traveled in the platform quadrant in the MWM test. Moreover, the two groups showed no significant difference in the swimming velocity (Figs. 7d and S7b). Immunofluorescence staining revealed enlarged lateral ventricle area (Figs. 7e and S7c), increased neuronal loss (Fig. S7d), and increased tauopathy (Fig. S7e) in TRPV1^−/−^/E4 (AAV-hTau) mice.

Microglia in TRPV1^−/−^/E4 (AAV-hTau) mice exhibited decreased process length, surface area, volume, and terminal and branching points (Fig. 7f, g). Consistent with GSEA analysis (Fig. S6f), large BODIPY^+^ neutral lipid inclusions were contained within microglia of TRPV1^−/−^/E4 (AAV-hTau) mice (Fig. 7h), and the percentage of BODIPY^+^ microglia was more than two-fold higher in TRPV1^−/−^/E4 (AAV-hTau) mice compared with E4 (AAV-hTau) mice (Fig. 7i). We further analyzed ER stress and SREBP2 activation in hippocampal microglia and observed that p-eIF2α (Ser 51) and SREBP2 expression was upregulated in the microglia of TRPV1^−/−^/E4 (AAV-hTau) mice compared with E4 (AAV-hTau) mice (Fig. 7j).

We examined the phagocytic capacity of hippocampal microglia and observed higher CD68 expression in microglia of TRPV1^−/−^/E4 (AAV-hTau) mice than E4 (AAV-hTau) mice. Consistently, the TRPV1^−/−^/E4 (AAV-hTau) mice showed increased microglial engulfment of extracellular tau and synaptic elements (Fig. S7f, g). These data together suggested that specific knockout of microglial TRPV1 exacerbates neurodegeneration and tauopathy in *APOE4* mice through modulating functional states and cholesterol metabolism of microglia.

Microglia expressing CCL8 and CD4 or CD8 T cells expressing CCR1 were markedly elevated in the hippocampus of TRPV1^−/−^/E4 (AAV-hTau) mice (Fig. 7k), which was consistent with the 116 upregulated genes enriched in BPs associated with chemokine ligand production and ECM organization (Fig. S6d). Flow cytometry indicated a dramatic increase in MHC II expression on resident microglia of TRPV1^−/−^/E4 (AAV-hTau) mice (Fig. 7l). We further examined T cell infiltration through flow cytometry, which showed a larger T cell fraction mainly comprising CD4 T cells in TRPV1^−/−^/E4 (AAV-hTau) mice than in E4 (AAV-hTau) mice (Fig. 7m). Immunostaining showed extensive microglial MHC II expression, T cell infiltration and interactions between microglia and CD4/CD8 T cells in the hippocampus in TRPV1^−/−^/E4 (AAV-hTau) mice (Fig. 7n). Overall, these studies demonstrated that microglia lacking TRPV1 have enhanced correlation with T cells in E4 mice with tauopathy.

## Discussion

In this study, we showed that *APOE4* accelerated neuronal perturbation and memory decline in a tauopathy mouse model, through increased MHC II-dependent antigen presentation within microglia and enhanced T cell priming and infiltration, as a consequence of cholesterol accumulation in microglia. *APOE4*-induced ER stress, specifically resulting from the ER Ca^2+^ depletion, promotes the SREBP2 transcriptional activation of the cholesterol biosynthesis pathway in microglia. Capsaicin reversed ER Ca^2+^ depletion, cholesterol accumulation, lysosomal dysfunction and immunophenotypic abnormality in ApoE4-treated microglia via modulating the Ca^2+^ influx. These results indicated that TRPV1 is a therapeutic target for restoring microglial cholesterol homeostasis for the treatment of AD.

AD is an age-associated neurodegenerative disorder characterized by symptoms such as memory impairment and pathologies including β-amyloid (Αβ) plaque deposition, neurofibrillary tangles and neuroinflammation [42]. Some well-recognized transgenic AD mouse models widely used in AD-related studies include APOE2, E3, E4 targeted replacement mice, PS19 mice, rTg4510 mice, 5 × FAD mice, APP/PS1 mice, and 3 × Tg AD mice [43]. Recent studies in AD mouse models have demonstrated that TRPV1 plays a protective role in AD-like pathophysiology, highlighting its potential as a therapeutic target for AD. TRPV1 activation through chronic dietary capsaicin decreases amyloid pathology and attenuates cognitive deficits in APP/PS1 mice [9, 44]. Daily intraperitoneal administration of capsaicin decreases Αβ plaque deposition and neurofibrillary tangles and improves spatial learning and memory in 3 × Tg AD mice [45]. More interestingly, genetically upregulating TRPV1 ameliorates deficits in hippocampal LTP and reverses memory impairments in APP23/PS45 mice [11]. In addition, TRPV1 agonist capsaicin rescues the ApoE4-induced disruption of intracellular lipid homeostasis and further diminishes synaptic phagocytosis by microglia in *APOE4* mice on a high-fat diet [5]. Here, we report that capsaicin administration reduced microglial cholesterol accumulation and improved cognitive function of *APOE*4 mice with tauopathy, while microglia‐specific TRPV1 deletion accelerated pathology in *APOE4* mice with tauopathy.

ApoE is a multifunctional protein that mediates cellular and systemic cholesterol metabolism in an isoform-dependent manner, mainly through binding lipoproteins, and is synthesized by astrocytes and activated microglia in the brain [46, 47]. Previous studies of ApoE as an immunomodulator in APCs, including dendritic cells [8, 47], macrophages [48, 49] and B cells [40], have shown that ApoE deficiency or the ApoE4 isoform enhances lipid raft enrichment, and provides a complex of immune cell-expressed surface molecules that are essential for optimizing T cell activation, which consists of MHC II molecules with antigen-derived peptides and co-stimulatory surface proteins. Here, abnormally aggregated highly phosphorylated tau protein was clearly present in microglia in the hippocampus of E4 (AAV-hTau) mice (Fig. 2d), accompanied by enhanced microglial antigen presentation via MHC II molecules and increased T cell activation and proliferation (Fig. 2f–h).

Localization and endocytosis of MHC I and MHC II molecules in immune cells have been shown to be associated with distinct types of membrane domains, and MHC II molecules show a strong preference for lipid rafts [8, 50]. Our observations indicated that MHC II molecules strongly clustered with lipid rafts in BV2 cells treated with ApoE4 in the presence of PHF (Fig. 4e, f). This suggested that ApoE4 may potentially compromise the integrity of blood–brain barrier (BBB) and allow the migration of peripheral immune cells, specifically CD4 T cells but not CD8 T cells, in the brains of AAV-hTau-injected E4 mice (Fig. 2g, h). The cytokine IL2, which is mainly expressed by activated CD4 T cells, plays a core role in T cell proliferation and activation of macrophages [51, 52]. Here, we found that *IL2Rβ* expression was significantly increased in BV2 cells treated with ApoE4 in the presence of PHF (Fig. 4i), and activation of TRPV1 downregulated the expression of *IL2Rβ* in microglia (Figs. 4i and S4c).

Notably, although the present study focused on CD4 T cell infiltration as a consequence of MHC II-dependent antigen presentation by microglia in AD, CD8 T cells have been shown to be expanded in neurological diseases [2, 30, 53]. We also observed an increased CD8 T cell population (Fig. 2g) in close association with microglia in the brains of AAV-hTau-injected E4 mice (Fig. 2h). A study using single-cell RNA and T cell receptor sequencing has revealed infiltration of CD8 T cells across the BBB, as mediated by microglia-derived chemokines in mice with radiation-induced brain injury [30]. Furthermore, ApoE4 and phosphorylated tau protein accelerate the development of AD through promoting the functional decline and the loss of integrity of the BBB [54, 55]. Our observations indicated a chemotactic axis through which the microglia-derived CCL8 chemokine mediates the infiltration of CCR1^+^ CD8 T cells in the brains of AAV-hTau-injected E4 mice (Fig. 2e), which is consistent with GSEA analysis in microglia from hiPSCs and mouse models (Figs. 2a, 6a, b, and 7a). Nonetheless, much more research is necessary to better understand the potential role of T cells in AD.

ER stress can lead to the release of Ca^2+^, thus modulating Ca^2+^-dependent functions [56, 57]. Lipid-free ApoE4 has a high propensity of misfolding and aggregation than other isoforms [58, 59], leading to an ER stress response in the brain [60]. Here, massive intracellular accumulation of ApoE protein in microglia was observed after incubation with ApoE4 and PHF, inducing ER stress and depletion of ER Ca^2+^ (Figs. 3i, j and S3a). Interestingly, compared with ApoE3, ApoE4 is associated with greater dysregulation of cholesterol and lipid metabolism in glia [5, 61]. ER stress is also known to stimulate the nuclear translocation of SREBP, which regulates the expression of genes involved in lipid synthesis [62]. Moreover, the ER Ca^2+^ level fine-tunes the ER retention capacity of SREBP2 [36]. In general, SREBP1 primarily regulates the expression of fatty acid triglyceride and phosphate synthesis genes, whereas SREBP2 primarily regulates expression of cholesterol biosynthesis genes [63]. SREBP2 accelerates key neuropathological hallmarks in AD and Huntington disease via cholesterol biosynthesis and transport, and cholesterol-mediated ROS accumulation [64, 65]. Consistently, we observed significant enrichment of genes associated with unfolded protein response and regulation of cholesterol biosynthesis by SREBP in *APOE4* microglia derived from hiPSCs of AD, with significantly increased expression of *SREBP2* (Fig. 1b–e).

Dysregulation of voltage-gated calcium channel activity and cytosolic calcium ion concentration in *APOE4* microglia provided a new insight into AD intervention (Fig. 3b). TRPV1 is a Ca^2+^-permeable cation channel that can be activated by temperature and various exogenous and endogenous stimuli, such as capsaicin, acid and ATP. TRPV1 is abundantly expressed in the CNS. Previous studies have reported that activation of TRPV1 with capsaicin regulates microglial energy metabolism, enhances microglial autophagy and phagocytosis, and inhibits M1 microglial polarization [9, 66, 67]. Here, the significant enrichment of gene sets associated with positive regulation of voltage-gated calcium channel activity, cellular response to calcium ion, ion homeostasis and cholesterol homeostasis in microglia from E4 (AAV-hTau) + CAP mice, further confirm TRPV1 as a novel therapeutic target for neurodegenerative disorders (Fig. 3c, e). Therefore, we focused on whether TRPV1 regulates SREBP2 activation by mediating transient Ca^2+^ influx. By using a Ca^2+^-sensitive fluorescent probe, we found that the TRPV1 agonist capsaicin significantly enhanced exogenous Ca^2+^ influx into the cytoplasm and decreased the depletion of ER Ca^2+^ in microglia (Fig. 3h). A part of TRPV1 locates on the ER; however, the sensitivity of ER-located TRPV1 to agonists is lower than the sensitivity of plasma membrane-located TRPV1, which is critical for cell health [68]. Our data strongly suggest that TRPV1 activation increases cytosolic and ER Ca^2+^ levels.

In summary, these findings indicate that TRPV1 is a potential therapeutic target connecting innate and adaptive immune responses for preventing neurodegeneration in AD.

## Conclusions

Collectively, we demonstrated that *APOE4* promotes cholesterol overload in microglia via the ER stress–ER Ca^2+^–SREBP2 pathway in response to tau pathogy. Cholesterol accumulation in microglia enhances MHC II-dependent antigen presentation and T cell infiltration. TRPV1-mediated Ca^2+^ influx in microglia reduces cholesterol accumulation and rescues neurodegeneration in *APOE*4 mice with tauopathy.

## Supplementary Information

**Additional file 1.** **Table S1** Primer sequences for Real-time PCR. **Figure S1 **APOE4 disturbs microglial cholesterol transport in human AD and exacerbates hTau-induced neurodegeneration. **Figure S2** Flow cytometric gating strategy. **Figure S3** TRPV1 agonist promotes cholesterol efflux and rescues immunophenotypic switch in ApoE4 + PHF microglia. **Figure S4** Administration of the TRPV1 agonist ameliorates microglial antigen presentation and T cell infiltration in E4 mice with tauopathy. **Figure S5** Administration of the TRPV1 agonist rescues hTau-induced neurodegeneration, tauopathy and microglial phagocytic dysfunction in E4 mice. **Figure S6** Microglia-specific TRPV1 deficiency disturbs cholesterol homeostasis and exacerbates MHC II-antigen presentation of microglia in E4 mice with tauopathy. **Figure S7 **Microglia-specific TRPV1 deficiency exacerbates hTau-induced memory deficits, tauopathy and microglial phagocytic dysfunction in E4 mice.

## Data Availability

The datasets used and analyzed during the current study are available from the corresponding author on reasonable request.

## Abbreviations

AD: Alzheimer’s disease
ApoE: Apolipoprotein E
APOE4: ε4 allele of *APOE* gene
TRPV1: Transient receptor potential vanilloid type 1
hiPSCs: Human induced pluripotent stem cells
MHC II: Major histocompatibility complex II
ER: Endoplasmic reticulum
SREBP2: Sterol regulatory element-binding protein 2
TEM: Transmission electron microscopy
PHF: Paired helical filament
ROS: Reactive oxygen species
GSEA: Gene set enrichment analysis
AAV-GFP: Adeno-associated virus for generating N-terminal protein fusion with green fluorescent protein
AAV-hTau: Adeno-associated virus encoding human full-length tau
MWM: Morris water maze
p-eIF2α (Ser 51): Phosphorylated eukaryotic initiation factor-2α
FBS: Fetal bovine serum
GRP78: Glucose regulated proteins 78
KEGG: Kyoto Encyclopedia of genes and genomes
GO: Gene ontology
ECM: Extracellular matrix
BBB: Blood–brain barrier
