# Supplementary material for: TRPV1 alleviates APOE4-dependent microglial antigen presentation and T cell infiltration in Alzheimer's disease
Source: Transl Neurodegener. 2024 Oct 29;13:52. doi: 10.1186/s40035-024-00445-6 (PMC11520887; doi:10.1186/s40035-024-00445-6)
Supplement: Supplementary file 1 — Additional file 1. Table S1 Primer sequences for Real-time PCR. Figure S1 APOE4 disturbs microglial cholesterol transport in human AD and exacerbates hTau-induced neurodegeneration. Figure S2 Flow cytometric gating strategy. Figure S3 TRPV1 agonist promotes cholesterol efflux and rescues immunophenotypic switch in ApoE4 + PHF microglia. Figure S4 Administration of the TRPV1 agonist ameliorates microglial antigen presentation and T cell infiltration in E4 mice with tauopathy. Figure S5 Administration of the TRPV1 agonist rescues hTau-induced neurodegeneration, tauopathy and microglial phagocytic dysfunction in E4 mice. Figure S6 Microglia-specific TRPV1 deficiency disturbs cholesterol homeostasis and exacerbates MHC II-antigen presentation of microglia in E4 mice with tauopathy. Figure S7 Microglia-specific TRPV1 deficiency exacerbates hTau-induced memory deficits, tauopathy and microglial phagocytic dysfunction in E4 mice. [file 40035_2024_445_MOESM1_ESM.docx]

**﻿Supporting Information**

**Table S1** Primer sequences for Real-time PCR.

| Primer | Forward (5'-3') | Reverse (5'-3') |
| --- | --- | --- |
| *SREBP2* | GCAGCAACGGGACCATTCT | CCCCATGACTAAGTCCTTCAACT |
| *HMGCR* | AGCTTGCCCGAATTGTATGTG | TCTGTTGTGAACCATGTGACTTC |
| *HMGCS1* | GGAAGCCTTTGGGGACGTTA | ACACTCCAACCCTCTTCCCT |
| *SQLE* | CTTCTCCTCAAAGCGAGCACA | TTATTTAAAAATCGCCTGCTGGA |
| *PLIN1* | GGGACCTGTGAGTGCTTCC | GTATTGAAGAGCCGGGATCTTTT |
| *H2Aa* | CTGACCACCATGCTCAGCCTCT | TACTGGCCAATGTCTCCAGGAG |
| *CD74* | GGATGGCGTGAACTGGAAGA | CCTGGCACTTGGTCAGTACTTT |
| *IL10* | CGTTGGGAATGGCTATGTC | GAGGCCCTGGCTGAGTAA |
| *TNF-α* | CAGGAGGGAGAACAGAAACTCCA | CCTGGTTGGCTGCTTGCTT |
| *IL-1β* | TCCAGGATGAGGACATGAGCAC | GAACGTCACACACCAGCAGGTTA |
| *Ifit1* | CTGAGATGTCACTTCACATGGAA | GTGCATCCCCAATGGGTTCT |
| *Mx1* | GACCATAGGGGTCTTGACCAA | AGACTTGCTCTTTCTGAAAAGCC |
| *CCL8* | TCTACGCAGTGCTTCTTTGCC | AAGGGGGATCTTCAGCTTTAGTA |
| *IL2Rβ* | GTGGACCTCCTTGACATA | GTTTCGTTGAGCTTTGACCCTCA |
| *TNFRSF11α* | AGAAGCACACCAGGGGACAAC | ACAGAGATGAAGAGGAGCAGAACG |
| *GAPDH* | TTGATGGCAACAATCTCCAC | CGTCCCGTAGACAAAATGGT |

**
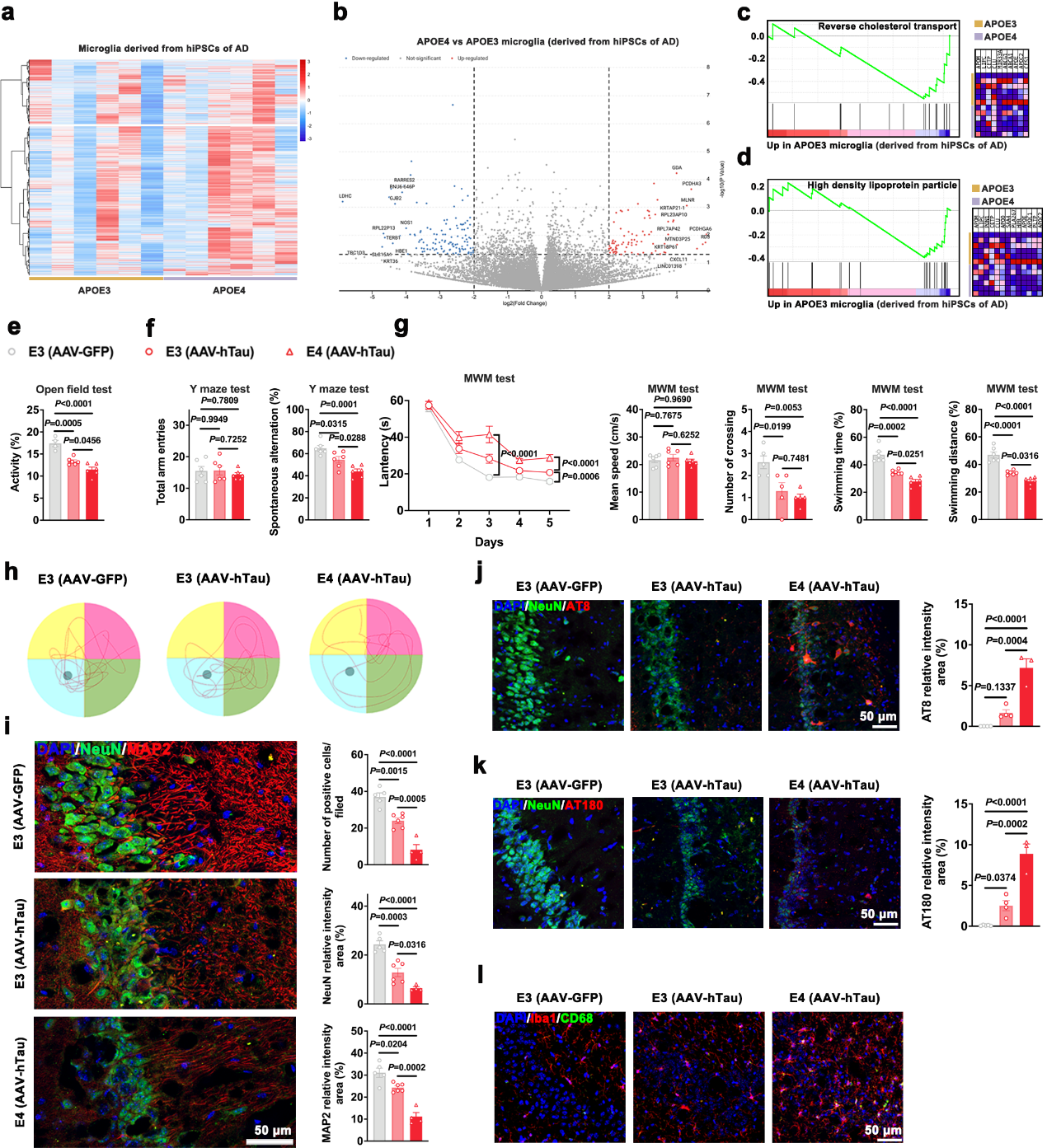
**

**Figure S1** APOE4 disturbs microglial cholesterol transport in in human AD and exacerbates hTau-induced neurodegeneration. Related to Figure 1. **a**, **b** Hierarchical clustering heatmap and volcano plot of gene expression changes in APOE4 microglia compared with APOE3 microglia (*n*=6) derived from hiPSCs of AD. **c**, **d** GSEA of reverse cholesterol transport and high-density lipoprotein particle pathway in microglia. **e** Open field test for E3 and E4 mice after bilateral intrahippocampal injection of AAV-GFP or AAV-hTau. Age-matched AAV-GFP-injected E3 mice (*n*=4), AAV-hTau-injected E3 mice (*n*=6) and AAV-hTau-injected E4 mice (*n*=6) are shown. **f** Total arm entries and spontaneous alternation in the Y maze test (*n*=6). **g** Escape latency during 5 consecutive days training period, swimming velocity, number of crossings, percentage of distance travelled and time spent in platform quadrant assessed by removing the platform in the MWM test at day 6 (*n*=5-6). **h** Representative tracks of mice swimming for E3 and E4 mice after bilateral intrahippocampal injection of AAV-GFP or AAV-hTau. **i** Representative immunofluorescence staining and quantification of NeuN (green) and MAP2 (red) in the hippocampus of mice (*n*=4-6 fields from 3 mice in each group). Scale bar, 50 μm. **j**, **k** Representative immunofluorescence staining and quantification of NeuN (green) and AT8 (red) (J) or AT180 (red) (K) in the hippocampus of mice (*n*=3-5 fields from 3 mice in each group). Scale bar, 50 μm. **l** Representative images of double immunofluorescence staining for Iba1 (red) and CD68 (green). Nuclei were counterstained with DAPI (blue). Scale bar, 50 μm or 10 μm. Statistical analysis among multiple groups were compared using one-way ANOVA with Tukey’s multiple comparisons test. Data presented are mean ± SEM.


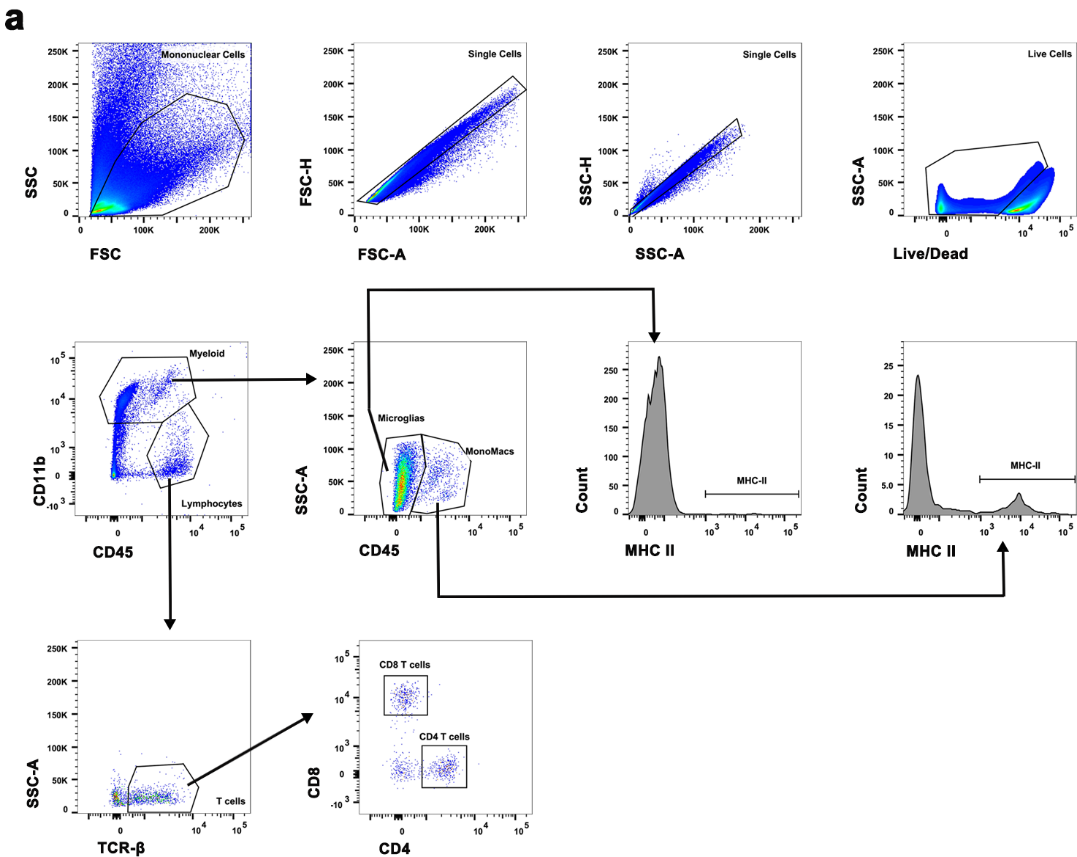


**Figure S2** Flow cytometric gating strategy. Related to Figure 2. **a** A representative flow cytometric gating strategy for identification of antigen presentation and T cells infiltration in hippocampus and cortex tissues of mice after bilateral intrahippocampal injection of AAV-GFP or AAV-hTau.

**
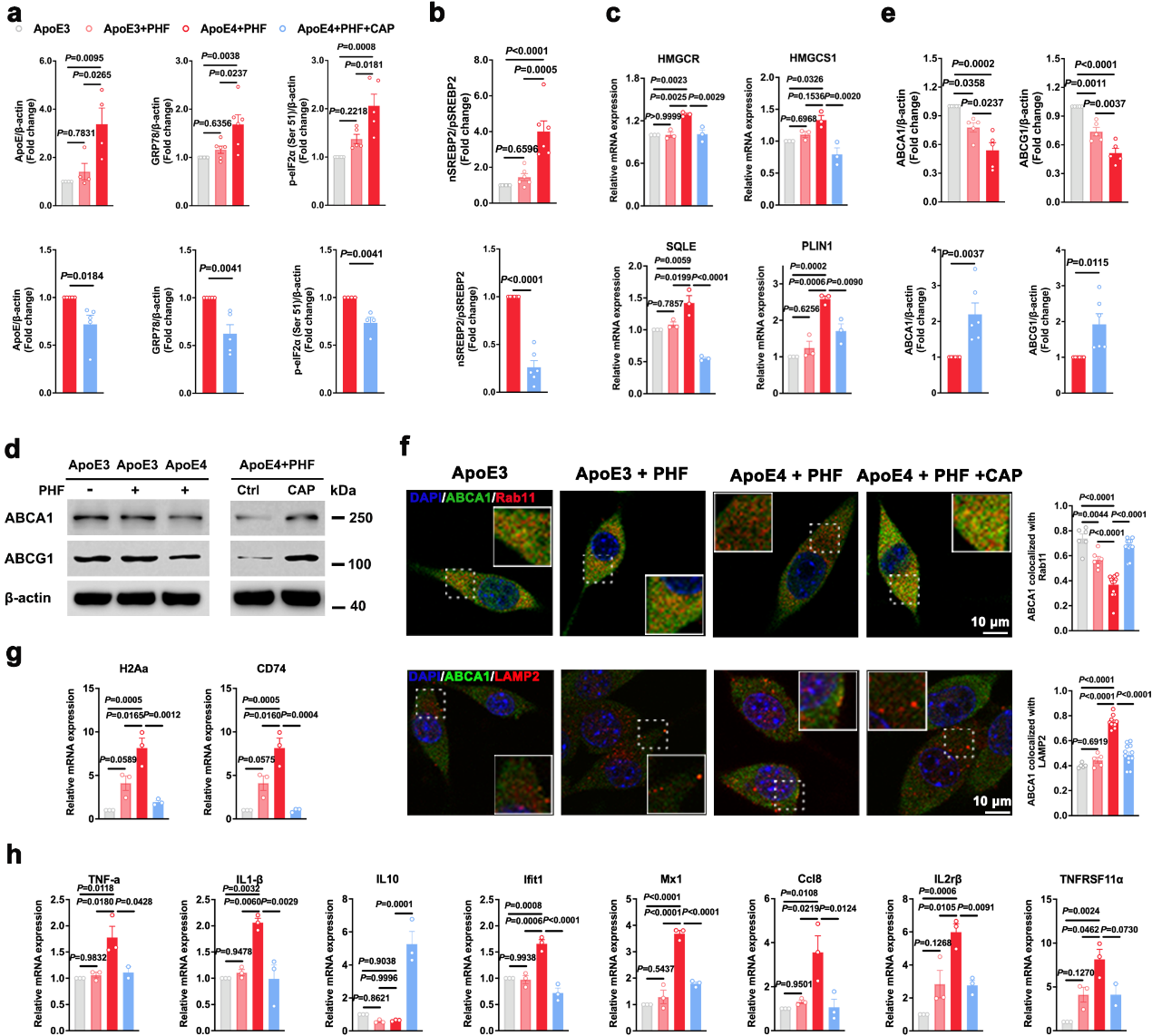
**

**Figure S3** TRPV1 agonist promotes cholesterol efflux and rescues immunophenotypic switch in ApoE4 + PHF microglia. Related to Figure 3 and Figure 4. **a** Quantification of ApoE, GRP78 and p-eIF2α (Ser 51) in ApoE4 + PHF BV2 cells after exposure to vehicle or capsaicin. ApoE (*n*=4 or 5, biological replicates), GRP78 (*n*=5 or 6, biological replicates) and p-eIF2α (Ser 51) (*n*=4 or 5, biological replicates). **b** Quantification of pre-mature SREBP2 and the activated nuclear SREBP2 (*n*=6, biological replicates). **c** Relative mRNA expression of cholesterol synthesis-related genes (*HMGCR*, *HMGCS1*, *SQLE* and *PLIN1*) (*n*=3, biological replicates). **d, e** Immunoblots (**d**) and quantification (**e**) of ABCA1 and ABCG1 (*n*=5 or 6, biological replicates). **f** Representative immunofluorescence staining and statistical analysis for the colocalization of ABCA1 signal (green) within Rab11 (red) or LAMP2 (red) in BV2 cells (*n*=5-13 fields from 3 biological replicates). **g, h** Relative mRNA expression of antigen presentation-related (*H2Aa* and *CD74*) (**g**) and inflammatory genes (*IL10*, *TNF-α*, *IL-1β*, *Ifit1*, *Mx1*, *CCL8*, *IL2Rβ* and *TNFRSF11α*) (**h**) in BV2 cells (*n*=3, biological replicates). Statistical analysis among multiple groups were compared using one-way ANOVA with Tukey’s multiple comparisons test. Statistical analysis between two groups was conducted using unpaired t-test. Data presented are mean ± SEM.

**
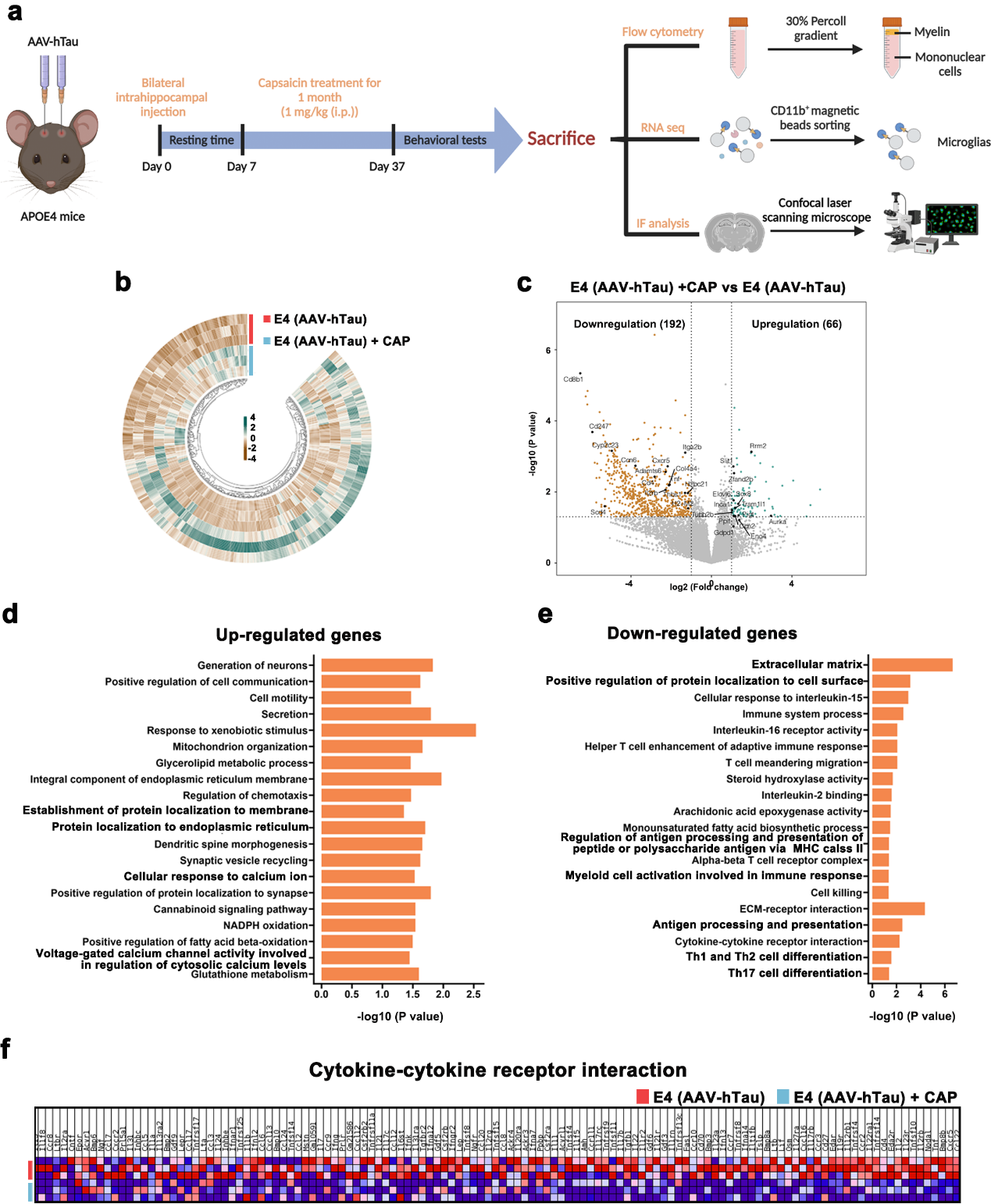
**

**Figure S4** Administration of the TRPV1 agonist ameliorates microglial antigen presentation and T cell infiltration in E4 mice with tauopathy. Related to Figure 6. **a** Schematic of capsaicin treatment and experimental analysis. **b** Two-way hierarchical heatmap of differential gene cluster analysis in microglia isolated from the hippocampus and cortex of 4-months old mice (*n*=3) from E4 (AAV-hTau) or E4 (AAV-hTau) + CAP mice. **c** A volcano plot of gene expression changes in microglia from E4 (AAV-hTau) + CAP mice compared with those in E4 (AAV-hTau) mice. **d, e** 20 enriched BPs were found among 66 upregulated genes (**d**) or 192 downregulated genes (**e**) in microglia from E4 (AAV-hTau) + CAP mice compared with those in E4 (AAV-hTau) mice. **f** Gene expression heatmap of cytokine-cytokine receptor interaction in microglia isolated from the hippocampus and cortex of 4-months old mice from E4 (AAV-hTau) or E4 (AAV-hTau) + CAP mice (*n*=3).


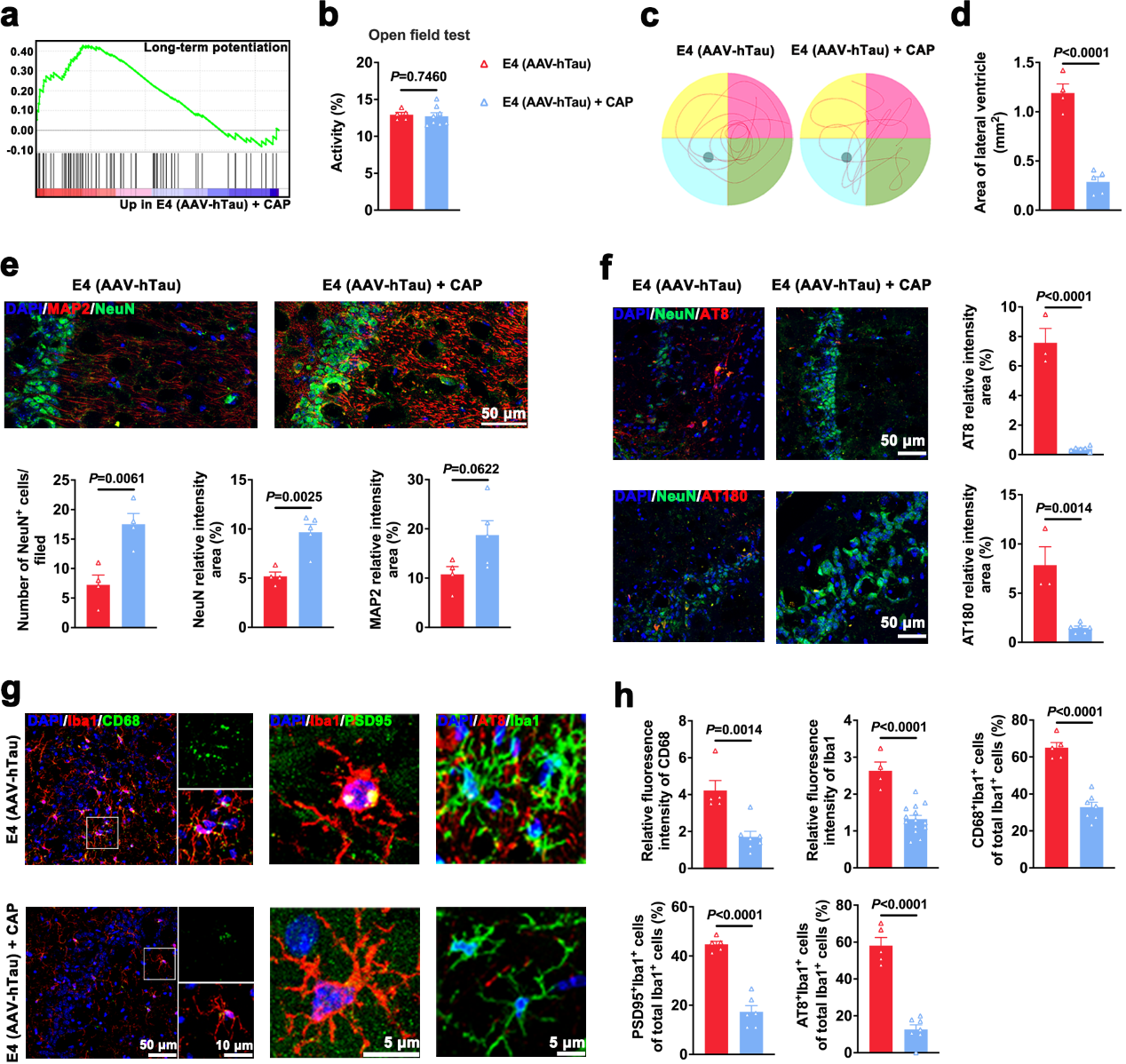


**Figure S5** Administration of the TRPV1 agonist rescues hTau-induced neurodegeneration, tauopathy and microglial phagocytic dysfunction in E4 mice. Related to Figure 6. **a** GSEA of long-term potentiation in microglia isolated from the hippocampus and cortex of 4-months old mice from E4 (AAV-hTau) or E4 (AAV-hTau) + CAP mice (*n*=3). **b** Open field test for E4 (AAV-hTau) mice treated with capsaicin. Age-matched E4 (AAV-hTau) mice (*n*=5) and E4 (AAV-hTau) + CAP mice (*n*=8) are shown. **c** Representative tracks of mice swimming. **d** Quantification of the area of lateral ventricle of mouse brain sections (*n*=4 or 5 fields from 3 mice in each group). **e** Representative immunofluorescence images and quantification of NeuN (green) and MAP2 (red) in the hippocampus of mice (*n*=4 or 5 fields from 3 mice in each group). Scale bar, 50 μm. **f** Representative immunofluorescence staining and quantification of NeuN (green) and AT8 (red) or AT180 (red) in the hippocampus of mice (*n*=3-6 fields from 3 mice in each group). Scale bar, 50 μm. **g, h** Representative immunofluorescence images and quantification of Iba1 (red) and CD68 (green), Iba1 (red) and PSD95 (green) or Iba1 (green) and AT8 (red) in the hippocampal CA3 of E4 (AAV-hTau) mice treated with capsaicin (*n*=4-14 fields from 3 mice in each group). Scale bar, 50 μm or 10 μm. Nuclei were counterstained with DAPI (blue). Statistical analysis between two groups was conducted using unpaired t-test. Data presented are mean ± SEM.


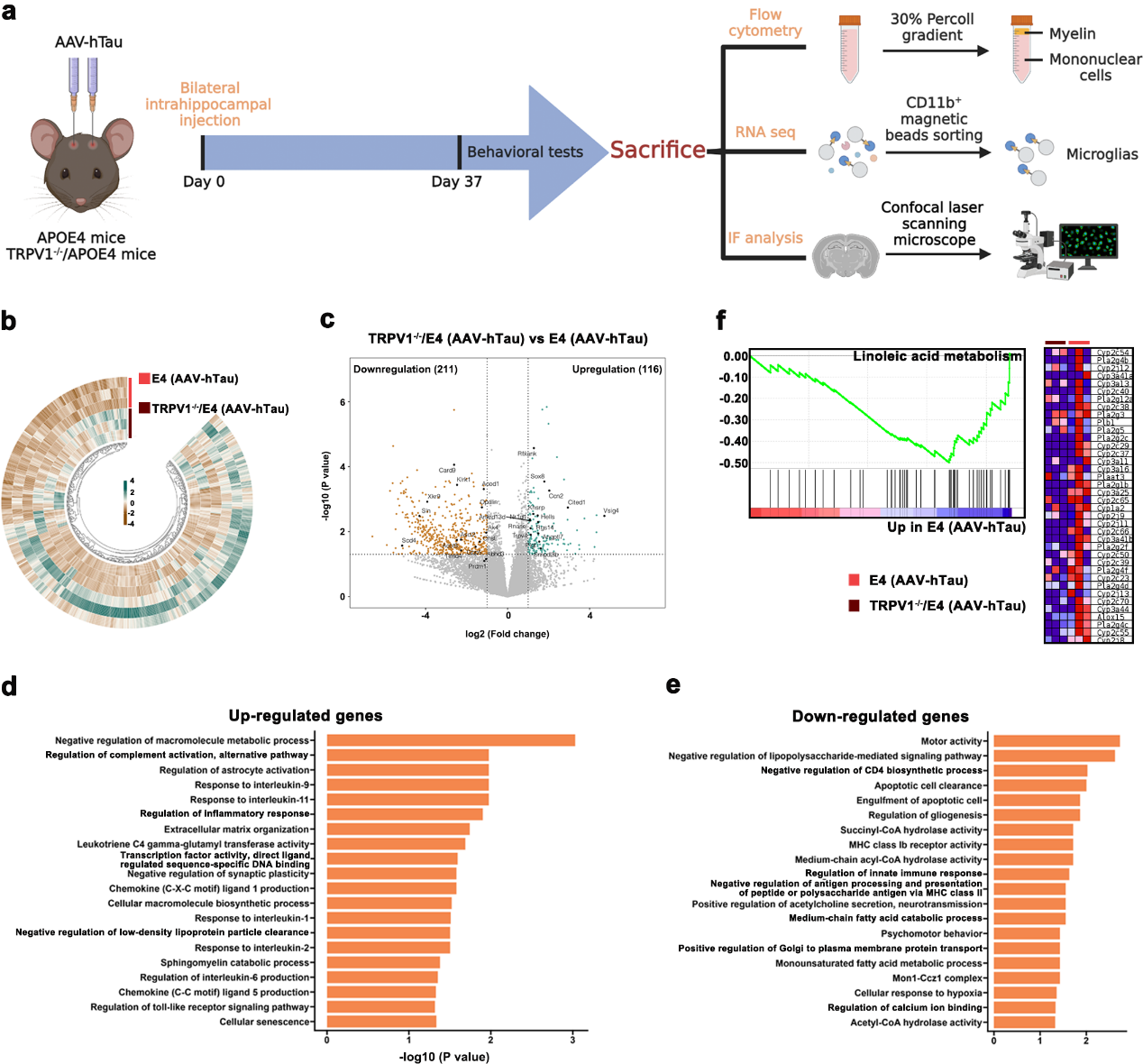


**Figure S6** Microglia-specific TRPV1 deficiency disturbs cholesterol homeostasis and exacerbates MHC II-antigen presentation of microglia in E4 mice with tauopathy. Related to Figure 7. **a** A schematic diagram illustrating the experimental analysis. **b** Two-way hierarchical heatmap of differential gene cluster analysis of microglia isolated from the hippocampus and cortex of 4-months old mice from E4 (AAV-hTau) or TRPV1^-/-^/E4 (AAV-hTau) mice (*n*=3). **c** A volcano plot of gene expression changes in microglia from TRPV1^-/-^/E4 (AAV-hTau) mice compared with those in E4 (AAV-hTau) mice. **d, e** 20 enriched BPs were found among 116 upregulated genes (**d**) or 211 downregulated genes (**e**) in microglia from TRPV1^-/-^/E4 (AAV-hTau) mice compared with those in E4 (AAV-hTau) mice. **f** GSEA and gene expression heatmap of linoleic acid metabolism in microglia isolated from the hippocampus and cortex of 4-months old mice from E4 (AAV-hTau) or TRPV1^-/-^/E4 (AAV-hTau) mice (*n*=3).


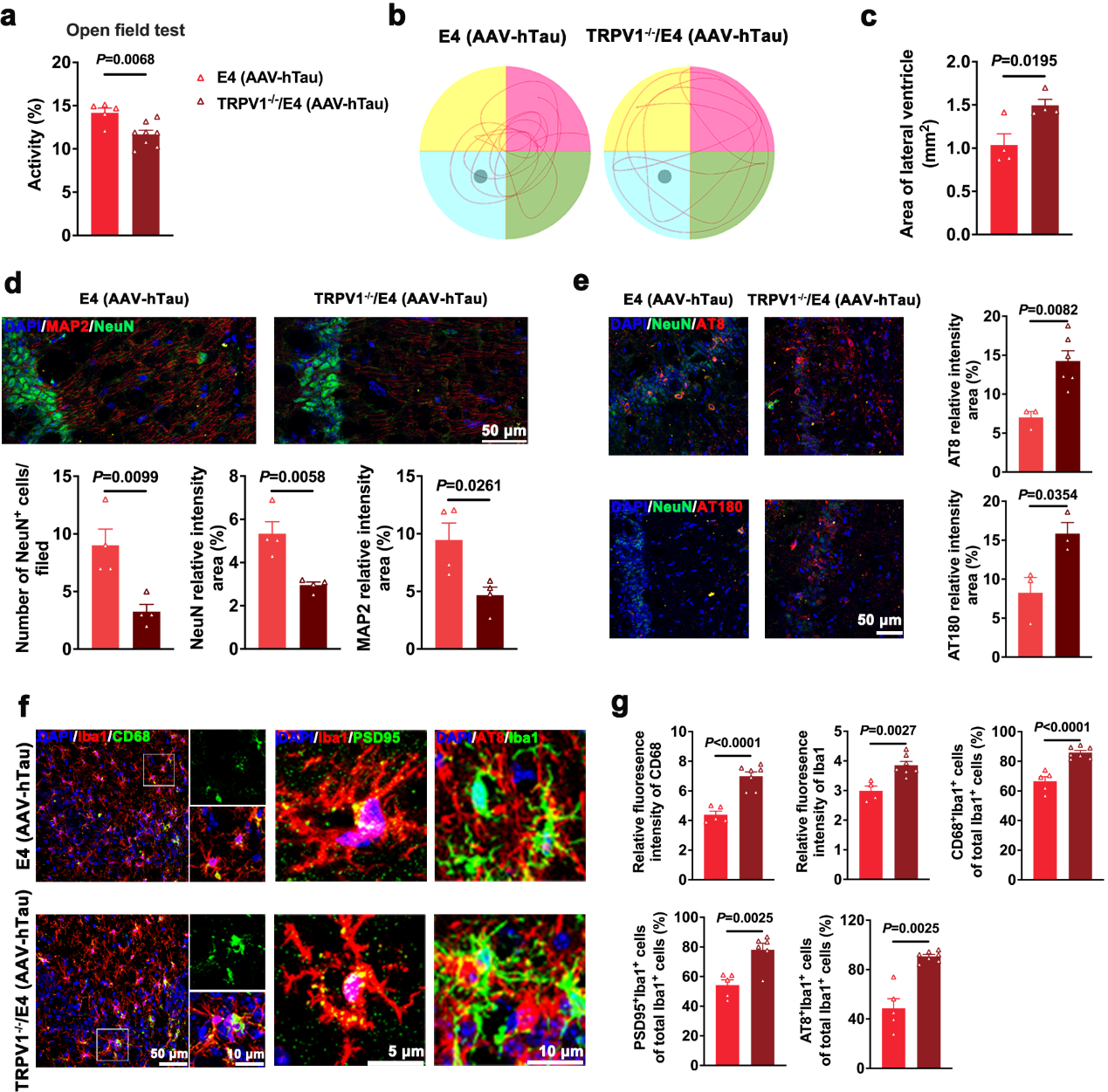


**Figure S7** Microglia-specific TRPV1 deficiency exacerbates hTau-induced memory deficits, tauopathy and microglial phagocytic dysfunction in E4 mice. Related to Figure 7. **a** Open field test. Age-matched E4 (AAV-hTau) mice (*n*=5) and TRPV1^-/-^/E4 (AAV-hTau) mice (*n*=8) are shown. **b** Representative tracks of mice swimming. **c** Quantification of the area of lateral ventricle (*n*=4 fields from 3 mice in each group). **d** Representative staining and quantification of NeuN (green) and MAP2 (red) (*n*=4 fields from 3 mice in each group). Scale bar, 50 μm. **e** Representative images and quantification of NeuN (green) and AT8 (red) or AT180 (red) (*n*=3-6 fields from 3 mice in each group). Scale bar, 50 μm. **f**, **g** Representative immunofluorescence images (**f**) and quantification (**g**) of Iba1 (red) and CD68 (green), Iba1 (red) and PSD95 (green) or Iba1 (green) and AT8 (red) in the hippocampal CA3 of TRPV1^-/-^/E4 (AAV-hTau) mice (*n*=4-7 fields from 3 mice in each group). Scale bar, 50 μm or 10 μm. Nuclei were counterstained with DAPI (blue). Statistical analysis between two groups was conducted using unpaired t-test. Data presented are mean ± SEM.
